# Free Energy Perturbation Calculation of Relative Binding Free Energy between Broadly Neutralizing Antibodies and the gp120 Glycoprotein of HIV-1

**DOI:** 10.1016/j.jmb.2016.11.021

**Published:** 2017-04-07

**Authors:** Anthony J. Clark, Tatyana Gindin, Baoshan Zhang, Lingle Wang, Robert Abel, Colleen S. Murret, Fang Xu, Amy Bao, Nina J. Lu, Tongqing Zhou, Peter D. Kwong, Lawrence Shapiro, Barry Honig, Richard A. Friesner

**Affiliations:** 1 Department of Chemistry, Columbia University, 3000 Broadway, MC 3178, New York, NY 10027, USA; 2 Department of Biochemistry and Biophysics, Columbia University Medical Center, 701 West 168th Street, New York, NY 10032, USA; 3 Vaccine Research Center, NIAID, National Institutes of Health, 40 Convent Drive, Bethesda, MD 20892, USA; 4 Schrodinger Inc., 120 W 45th Street, New York, NY 10036, USA; 5 Department of Biochemistry and Molecular Biophysics, Center for Computational Biology and Bioinformatics, Department of Systems Biology, Department of Medicine, Howard Hughes Medical Institute, Columbia University, 1130 Street Nicholas Avenue, Room 815, New York, NY 10032, USA; 6 Department of Pathology, Columbia University Medical Center, 630 W. 168th St, New York, NY 10032, USA

**Keywords:** bNAbs, broadly neutralizing antibodies, CDR H2, second heavy chain complementarity-determining region, FEP, free energy perturbation, MD, molecular dynamics, GPU, graphics processing unit, RMSE, RMS error, RSC3, resurfaced stabilized core 3, PDB, protein data bank, PB, Poisson–Boltzmann, REST, replica exchange solute tempering, computational chemistry, binding affinity optimization, physics-based models, alchemical FEP, protein structure prediction

## Abstract

Direct calculation of relative binding affinities between antibodies and antigens is a long-sought goal. However, despite substantial efforts, no generally applicable computational method has been described. Here, we describe a systematic free energy perturbation (FEP) protocol and calculate the binding affinities between the gp120 envelope glycoprotein of HIV-1 and three broadly neutralizing antibodies (bNAbs) of the VRC01 class. The protocol has been adapted from successful studies of small molecules to address the challenges associated with modeling protein–protein interactions. Specifically, we built homology models of the three antibody–gp120 complexes, extended the sampling times for large bulky residues, incorporated the modeling of glycans on the surface of gp120, and utilized continuum solvent-based loop prediction protocols to improve sampling. We present three experimental surface plasmon resonance data sets, in which antibody residues in the antibody/gp120 interface were systematically mutated to alanine. The RMS error in the large set (55 total cases) of FEP tests as compared to these experiments, 0.68 kcal/mol, is near experimental accuracy, and it compares favorably with the results obtained from a simpler, empirical methodology. The correlation coefficient for the combined data set including residues with glycan contacts, *R*^2^ = 0.49, should be sufficient to guide the choice of residues for antibody optimization projects, assuming that this level of accuracy can be realized in prospective prediction. More generally, these results are encouraging with regard to the possibility of using an FEP approach to calculate the magnitude of protein–protein binding affinities.

## Highlights

• Natural bNAbs against HIV-1 are a promising starting point for therapies.
• FEP provides a promising technique to computationally predict antibody structure optimizations.
• We show that FEP applied to bNAb mutations predicts binding affinity changes.
• FEP can provide an efficient method to identify potency-enhancing mutations to bNAbs.

## Graphical Abstract

**Figure:**
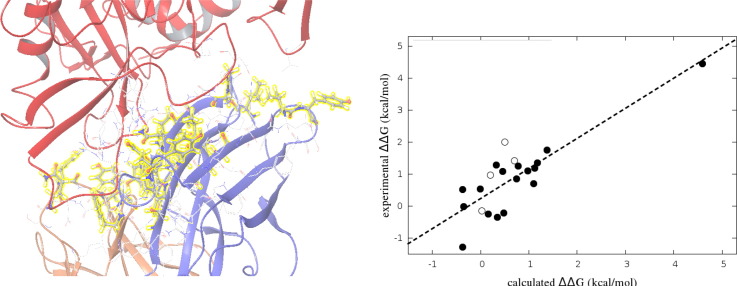

## Introduction

Over the past several years, there has been great interest in the use of broadly neutralizing antibodies (bNAbs) for both treatment and prophylaxis against HIV-1 infection [1], [2], [3], [4], [5], [6]. About 50% of HIV-1-infected individuals produce antibodies with considerable neutralization breadth for a wide range of circulating HIV-1 strains after about 5 years of chronic infection [1], [2], [7], [8]. However, as isolated from infected donors, most bNAbs would require high dosing to achieve efficacy. For example, human studies with the VRC01 antibody, which neutralizes ~ 90% of tested HIV-1 strains, utilized a minimal therapeutic dosage of 5 mg/kg of body weight [9]. Nevertheless, bNAbs represent a useful starting point for the development of suitable therapeutic and prophylactic agents.

Numerous HIV-1-neutralizing antibodies, including VRC01 [10], are directed against the gp120 surface glycoprotein, the major cell surface extracellular component of the HIV viral spike [3]. VRC01 is directed against the initial binding surface on gp120 recognized by the CD4 receptor on human host cells [11], a well-characterized site of HIV-1 vulnerability to antibody-mediated neutralization [12]. In VRC01, the recognition of the CD4-binding site is dominated by the antibody second heavy chain complementarity-determining region (CDR H2) [11]. VRC01 is the founding member of a class of related antibodies, the VRC01 class [13], which is characterized by the precise targeting of CD4-binding site [11], genetic signatures including its derivation from the VH1‐2 heavy chain gene, CDR L3 loops restricted to 5 aa in length, and high levels of somatic hypermutation (> 30%) for effective neutralizers [4], [13]. VRC01-class antibodies have been found in numerous donors with broadly neutralizing sera, and the recognition of gp120 by antibodies from diverse VRC01-class donors is similar [13].

Over the past several years, there have been extensive efforts to optimize antibody potency for a number of bNAbs [14], [15], [16]. While experimental optimizations of antibodies can examine a large number of potential candidates, they lack the flexibility to efficiently perform a wide exploration of possible bNAb mutants. Computational approaches can in principle complement experimental efforts by allowing an investigation of diverse combinations of mutation sites. For computation to make a useful impact, however, a high degree of accuracy and reliability in binding affinity prediction, coupled with a tractable computational cost, are required. Given the complexity of the gp120/antibody interface, this appears to be a daunting task, which is possibly outside of the realm of feasibility for the current state-of-the-art computational chemistry technology.

All-atom, explicit solvent-based free energy perturbation (FEP) methods [17], [18], employing molecular dynamics (MD) simulation methodology, constitute the most rigorous physics-based approach to the computation of binding free energies in complex biological systems. Historically, it has been difficult to perform accurate, converged FEP simulations, due to inadequate computing power, errors in molecular mechanics force fields, and inability of sampling algorithms to escape from local minima [19], [20], [21]. Over the past decade, these problems have been effectively addressed: the first by Moore's law and the advent of the use of graphics processing units (GPUs) for computation, the second by major improvements in force field quality, and the third by advanced sampling algorithms such as the replica exchange solute tempering (REST) method, which employs a version of local heating to enhance sampling in the region of the perturbation [17], [22], [23]. In a recent publication, we have shown that for the binding of small-molecule ligands to a variety of pharmaceutically interesting target proteins, our current FEP implementation is capable of achieving an RMS error (RMSE) on the order of 1 kcal/mol using a tractable amount of GPU resources [18]. These results have established FEP as a practical methodology for structure-based drug discovery projects.

While the use of FEP to assess protein–small-molecule ligand binding affinities has a long history, there have been relatively few attempts to apply the methodology to the calculation of protein–protein binding [24], [25], [26]. The interface between two proteins is typically larger and significantly more complex than that between a protein and a small-molecule ligand, suggesting that the sampling effort required to reliably converge such FEP calculations might well be substantially larger than that for protein–small-molecule binding. On the other hand, the results should depend only on the quality of the protein force field, so that the small-molecule ligand force field (for which it is quite challenging to attain robust coverage of chemical space) is eliminated as a possible source of error. On this basis, one might expect the modeling of protein–protein interactions via FEP to be a feasible undertaking, provided that the sampling challenges can be surmounted.

The objective of the present paper is to investigate the use of FEP methodology in modeling the binding of the VRC01-class bNAbs to gp120. The calculations were compared to alanine-scanning data in which the effects of antibody interface mutants on gp120 binding were quantified by surface plasmon resonance measurements. We attempt to predict the changes in binding free energy of antibody–gp120 complexes upon the mutation of the various interface residues of the antibody using FEP/REST. Three alanine scan data sets represent three initial antibodies from two different donors (VRC01 and VRC03 from NIAID donor 45 [10], and VRC-PG04 from IAVI donor 74 [4]). We consider all mutations studied experimentally other than those involving a net change in charge; one case is of a residue that is a terminus of its chain in the crystal structure used, and four proline mutants, which all were found to have experimental changes in binding affinity less than 0.5 kcal/mol in magnitude. Calculations on prolines would require significant technical effort to implement because of the need to alchemically change topologies from a ring structure to a linear backbone in such mutations, while mutations involving a change in net charge pose a significantly greater challenge to FEP than mutations that do not involve such a change. We and others have been developing methods to address changes in net charge [27], [28]; however, the methodology is still in the process of being tested. The total number of test cases, 55 in all, is sufficient to draw useful conclusions concerning performance.

Although the focus of the present effort is on optimizing and evaluating the FEP methodology, there are a number of other important issues that must be addressed if the system employed in the experiments is to be properly modeled. Firstly, while crystal structures exist for the antibodies in complex with gp120, the binding experiments were performed with a gp120 variant, which had a slightly different sequence than the one that was crystallized. This necessitated the construction of a homology model of the complex starting from the crystal structure. Secondly, the surface of the gp120 is decorated by numerous glycan residues, which are known to be important in the biological function of gp120 and in antibody binding. The characterization of the general glycan content has seen some recent progress [29], [30], but the precise chemical composition of the glycans in the experimental system used to measure binding affinity is not known. In an attempt to take glycans into account, we used the fragment of one particularly important glycan, NAG776, in the binding region observed in the crystal structure of the gp120 proteins used in building the homology models employed in this study. Inclusion of NAG776 is critical in obtaining the accurate prediction of free energy changes for a subset of mutations.

Once these two issues are addressed, the major remaining challenge is the modification of the standard sampling algorithm developed for small molecules so that it can handle the protein–protein interface. In the current study, we found one principal requirement to be the use of greatly increased simulation times for tryptophan (trp) residues, which, when mutated to alanine, frequently induce relatively large changes in the protein structure due to their considerable bulk. A second problem is manifested when a glycine (gly) residue is mutated to alanine. In this case, the larger alanine residue can induce a nontrivial change in the loop on which the residue is located and/or in the surrounding side chains, and the MD algorithm may have difficulty overcoming the sampling barriers to properly explore alternate conformations. We address this problem by using continuum solvent-based loop prediction methods to generate predictions of the structure after mutation and then by employing these predictions as an initial guess in the FEP simulations.

From the above, it should be apparent that obtaining experimentally relevant results for the systems of interest represents a considerable modeling challenge, beyond simply deploying a standardized FEP protocol. We view the present work as an extensive exploration of the various dimensions of the problem, as opposed to a demonstration that a robust, automated protocol is in hand suitable for application to an arbitrary protein–protein interaction problem without modification. With that caveat, the results shown below are quite encouraging with regard to the application of FEP methods to the prediction of protein–antibody interactions and specifically to modeling bNAbs bound to gp120. Prospective results, in which the calculations are done prior to experimental measurements, will be required to draw stronger conclusions.

The paper is organized as follows. The Results section first presents the brief descriptions of the bNAbs that were studied, then experimental results for the three antibody/gp120 binding affinity data sets, followed by comparisons to results obtained with FEP. In addition to reporting FEP results run using both a baseline default protocol and a protocol including several improvements, we also present results using a simple empirical model, FoldX [31], which provides a reference point to assess the value of the more elaborate and computationally expensive FEP approach. The Discussion section considers the successes, failures, and uncertainties observed for the data sets investigated and outlines the efforts that will be needed to construct a robust and efficient general methodology for modeling protein–protein interactions. The Models and Methods section describes the models and methodology used to perform the calculations, including experimental techniques, homology model building, treatment of glycans, loop and side-chain predictions, and FEP protocols. Finally, in the Conclusion, we summarize our results and discuss future prospects of the approach.

## Results

### Test systems studied and experimental binding affinities

**Fig. 1:**
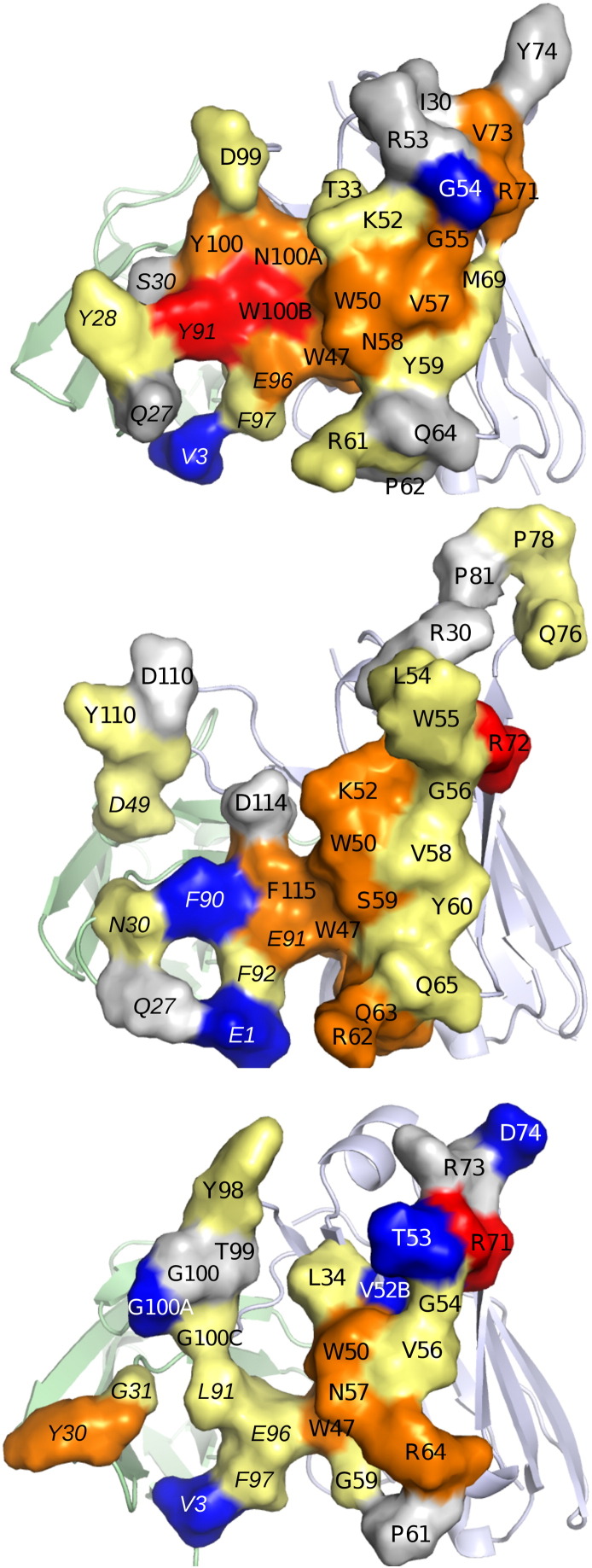
Antibody-gp120 interface residues of VRC01, VRC03, and VRC-PG04 antibodies. Antibody-binding interface is in surface representation with residues colored as follows: Red, ΔΔ*G* > 2 kcal/mol; Orange, ΔΔ*G* 1 to 2 kcal/mol; Yellow, ΔΔ*G* 0.5 to 1 kcal/mol; Gray, ΔΔ*G* − 0.5 to 0.5 kcal/mol; Blue, ΔΔ*G* <− 0.5 kcal/mol. Light chain residue numbers are italicized. Antibody non-interfacial residues are in cartoon representation colored pale blue (heavy chain) and green (light chain).

Three VRC01-class bNAbs were considered, for which experimental alanine scans with quantification of binding affinities using an Octet biosensor were performed (Table S1 in the Supplementary Data): VRC01 and VRC03 [10]—members of the same antibody lineage from NIAID donor 45—and VRC-PG04 from IAVI protocol G donor 74 [4]. Briefly, antibodies and mutants were bound to the Octet tip surface, and core gp120 were passed over the surface and sensograms recorded. K_d_s were determined by fitting to a 1:1 binding model (see Models and Methods). Estimated binding free energies typically fell within an uncertainty range of about 0.5 kcal/mol (see Table S2 of the Supplementary Data for details). Figure 1 presents all of the sites of mutation experimentally studied for each of the three antibodies listed above.

**Fig. 2:**
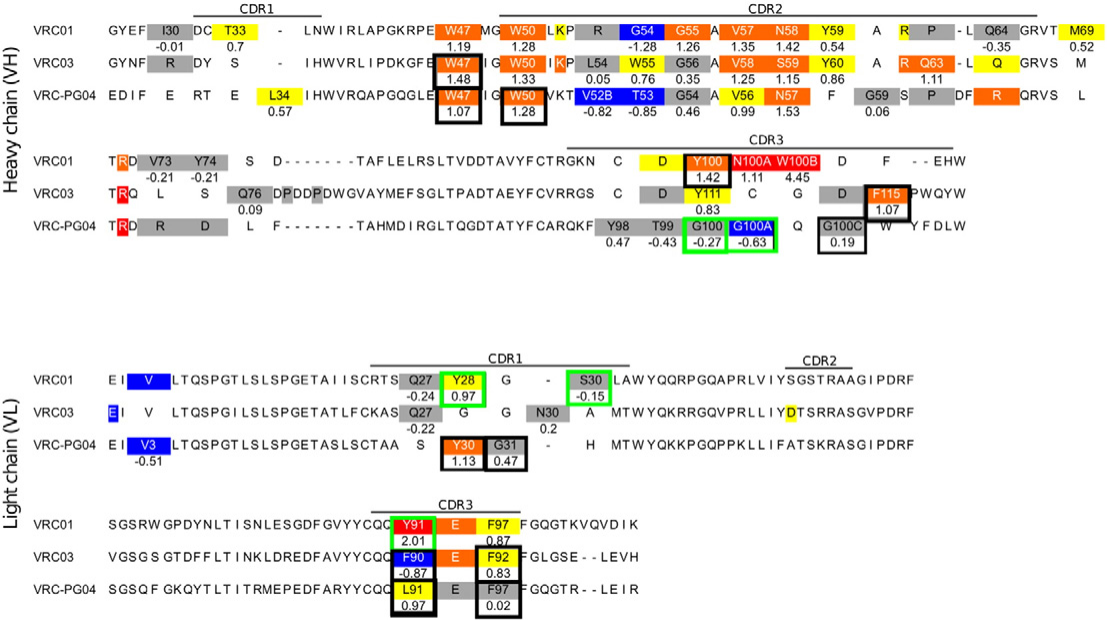
Sequence comparison for the heavy and light chains of the VRC01, VRC03, and VRC-PG04 antibodies. Alanine substitutions, with the resultant experimentally determined antibody gp120 binding free energy changes, are highlighted as follows: Red, ΔΔ*G* > 2 kcal/mol; Orange, ΔΔ*G* 1 to 2 kcal/mol; Yellow, ΔΔ*G* 0.5 to 1 kcal/mol; Gray, ΔΔ*G* − 0.5 to 0.5 kcal/mol; Blue, ΔΔ*G* <− 0.5 kcal/mol. ΔΔ*G* values for cases included in the test set are listed below each mutated residue. Strong and moderate glycan contact residues are boxed with black and green.

In this paper, we consider only the mutations of a neutral side chain to alanine. Figure 2 shows the experimentally determined ΔΔ*G* value for the 55 neutral residues included in the test set on the binding interface of the three antibodies. Most of these mutations lead to similar or weaker binding affinity, which is unsurprising given that for the most part, larger residues were mutated to alanine. A more extensive description of the experimental protocols, including error estimation, is provided in the Models and Methods section below.

### Model structures for the three experimentally relevant complexes

No crystal structures are available for these three antibodies in complex with the gp120 resurfaced stabilized core 3 (RSC3) [10] protein used in the Octet biosensor experiments we report. Therefore, a homology model of the RSC3 protein in complex with each antibody was constructed from an existing crystal structure for each antibody—the protein data bank (PDB) number **3NGB** for VRC01, and numbers **3SE8** and **3SE9** for VRC03 and VRC-PG04, respectively. Further details of the model are provided in the Models and Methods section. Aligned comparisons of the part of the three antibody sequences that contain residues that contact the gp120 protein in the bound complex are presented in Fig. 2. The structure of the antibody heavy and light chains is from the crystal structure in each case.

The gp120 glycoprotein is known to be heavily glycosylated, with glycosylation playing an important role in shielding HIV-1 from immune response [11], [29], [30], [32], [33], and in some cases, differences in glycan interactions between the wild-type and mutant residues may substantially affect the relative binding affinity arising from sequence mutations. Examination of the crystal structures identified one glycan fragment, residue *N*-acetylglucosamine (NAG776, attached to glycosylated residue N276), with direct interactions with residues in the binding interface in all three of the crystal structures. While some recent structures have been solved for bNAbs with the full glycan structure present [33], [34], [35], [36], [37], the structures for the bNAb–gp120 complexes used as templates in building homology models for the bNAbs in this study were solved using a truncated version of the glycan species (i.e., with a deglycosylated protein), so only a single asparagine-attached N-acetylglucosamine remained for each glycan. In contrast, experimental binding affinity measurements were performed using a fully glycosylated protein and so glycans had tails (containing additional sugar residues) attached to the moiety observable in the crystal structure. However, it is a reasonable approximation to assume that the interaction of the tail with the protein residues is minimal. However, the sugar moiety close to the surface represented by NAG776 in the crystal structure is likely to interact directly with the bound antibodies, and for this reason, we used the NAG776 core to account for glycan effects on binding.

NAG776 is sufficiently distant from many of the mutation sites that it is likely to have little or no impact on relative binding affinities. Other sites are much closer; in some cases, these are in direct contact with the glycan. We used a scoring function to classify each site into one of the three categories: close interaction, moderate interaction, and minimal interaction. The classification function, based on atomic contacts, is described in more detail in the Models and Methods section. Based on the contact scoring of the wild type system in a short MD simulation (see Models and Methods for further information), 11 residues were classified as having strong interactions (boxed in black in Fig. 1), 6 residues were classified to have moderate interactions (boxed in green in Fig. 1), and the remaining 38 were classified as having insignificant interactions. We investigated the effect of the presence of the glycan on the strong and moderate interaction cases; results are shown later in this section. We found that large effects were manifested only for strong interaction cases; for the 6 moderate interaction cases, the impact of the glycan on free energy changes was around 0.1 kcal/mol change in the RMSE, while the net effect on the RMSE for the 11 strong contact cases was more than 1 kcal/mol. Based on this evidence, we concluded that it was safe to ignore the glycan for the insignificant interaction cases and have done so in what follows.

### Default small-molecule FEP protocol

As an initial calibration point, we present results obtained from running the default FEP protocol utilized for small-molecule binding affinity prediction in Ref. [18], with a modestly increased simulation time to account for the significantly more complex interface structure. This protocol forms the starting point of our methodology; modifications designed to address specific problems found in default simulations are described in subsequent sections below. Details of the default protocol are given in the Models and Methods section.

In all cases shown here, only the side chain of the residue involved in the mutation was included in the REST region. Control simulations performed without REST on a significant subset of mutation cases indicated that the overall effects of this REST scheme in most of the alanine scan cases are small, but at least one case appeared to show significant improvement with REST (see Supplementary Data Table S4). Since there is negligible additional cost in simulation efficiency including REST over FEP alone, it was retained in all cases. Each calculation was repeated to reduce random noise arising from the Monte Carlo replica exchange algorithm. The OPLS3 protein force field was used for all simulations [38]. Recent tests, reported in Ref. [48], have shown that OPLS3 provides state-of-the-art performance with regard to protein and peptide stability. Furthermore, small-molecule FEP calculations have validated the ability of the force field to properly respond to ligand perturbation. We note that in our comparisons in Ref. [48], the CHARMm force field displayed a performance that was very similar to OPLS3 and thus would be likely to yield similar results to those presented below.

The homology models (see the Models and Methods section for further details) were used to carry out the calculations. We included the NAG776 glycan residue in the 17 cases identified as having close or moderate contacts with the residue being mutated, whereas it was not included for the remaining 38 cases. This aspect of the protocol was employed in all of the calculations that follow.

Table 1 displays the RMSEs and correlation coefficients, with the experimental results for the three data sets (VRC01, VRC03, and VRC-PG04) under the default protocol, and the largest outliers for each of these data sets. The overall results (particularly those for the VRC03 antibody) are significantly degraded from the most recent small-molecule data reported in Ref. [18]. Furthermore, assuming robust sampling, one would expect thatthe RMSE for protein–protein interactions should be smaller than for small-molecule perturbations, which additionally depend upon the quality of the small-molecule force field in representing highly diverse chemistry.

These results are not surprising, given the considerations discussed above. The default run time of 10 nsec (for each window in each FEP simulation leg) may not be adequate for all of the test cases, and some mutations may involve significant changes in loop geometry or local side-chain conformations, which necessitate measures going beyond longer run times in order to achieve a suitable transition from the initial to final loop structure. We conclude from this data that improvements in the sampling protocol are needed if the most quantitatively accurate results are to be obtained. In the following section, we outline several such improvements based on the analysis of the default protocol results.

### Extended sampling for Trp mutations and glycan contacts

Examination of the largest outliers in the default protocol results revealed that cases in which a tryptophan residue was mutated to alanine displayed errors much larger than the average. This observation led to the hypothesis that the sampling of Trp mutations may require significantly longer simulation time. As a simple test of this hypothesis, all tryptophan simulations were extended to 100 ns. To avoid complicating issues, we consider here the five TRP cases not identified as strong glycan contacts. Two independent 100 ns runs were averaged for these cases. The reduction of RMSE for this subset resulting from extending the simulation time was dramatic, from 1.85 kcal/mol to 0.97 kcal/mol. Results for individual cases can be found in Table 2.

**Fig. 3:**
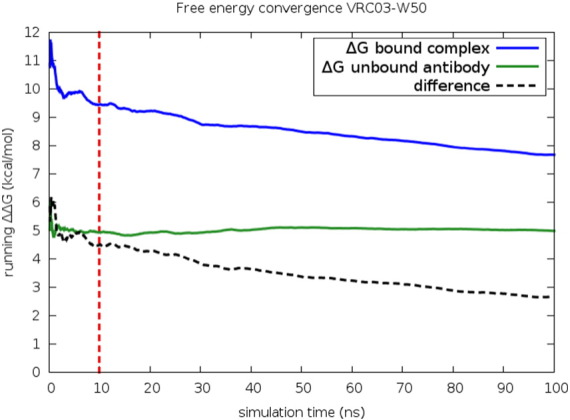
Running free energy for the W50 case on VRC03, averaged over two independent trials of both simulation legs. Slow convergence is observed in the bound complex leg.

**Fig. 4:**
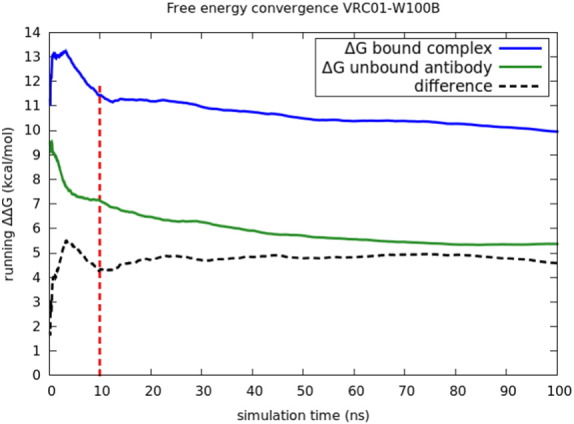
Running free energy for the W100B case on VRC01, averaged over two independent trials that were performed for this case. Slow convergence time scales are observed for both the bound and unbound legs of the simulation.

Running totals for the free energy change as a function of simulation time are displayed in Fig. 3, Fig. 4 for the example cases of the mutation of W50 in VRC03 and of W100B in VRC01. In four of the five TRP cases, the bound complex simulation leg shows a significant downward relaxation in predicted Δ*G* over the course of the run. The relaxation process is quite slow, and there can be some residual fluctuation in ΔΔ*G* at 100 nsec in some cases. However, the RMSE after 100 nsec, averaged over all cases, is comparable to that for the remainder of the data set, so extending the simulations further is unlikely to yield systematically better results, given the current force field quality and level of experimental error.

In the case of VRC01-W100B, the unbound (solvent) leg of the simulation also changed significantly with the longer simulation. The net result is that the longer simulations led to a comparable prediction as opposed to a smaller one. This was due to the removal of W100B allowing a large-scale rearrangement of the CDR H3 loop, which is at the interface of both the heavy chain and antigen and also the antibody heavy and light chains. This relaxation may take place on an even slower time scale than the 100-ns time scale can fully capture. It appears, however, that most of the fluctuation in ΔΔ*G* relaxed by 100 ns, and only the intra-antibody relaxation was continuing at 100 ns, making further extension unlikely to capture more net effect on the relative binding affinity. This case will be discussed in further detail in the subsection “Insights from FEP trajectories”.

We next investigated how the NAG776 glycan influenced the 17 cases identified above as having close or moderate contact with this moiety. Three sets of results are shown in Table 3 below. Firstly, we present the results of the default protocol for each of the 17 cases. Secondly, we present results in which the glycan is removed from the structure, still employing the default simulation protocol. Finally, results in which the glycan is retained, but the simulations are extended to 100 nsec, are given. The principal effects are seen for the strong contact cases; those with moderate contact evidence primarily relatively small fluctuations when the glycan is deleted or when the simulation extended. In the strong contact cases, both the glycan's presence and the extended simulation times were required to achieve good agreement with the experiment. Under these conditions, the RMSE of the full 17 contact set, 0.77, is only slightly larger than that of the non-contact cases (0.68 kcal/mol).

### Using loop prediction to generate an improved initial guess for additive mutations

Experience in using FEP for predicting small-molecule binding affinities has shown that the initial guess for the structure can have a significant impact on the final results. The natural initial guess used in the present case is the wild-type homology model, and that is what we have done in the default protocol. In most cases, this starting point yields reasonable results, suggesting that most mutations do not induce substantial conformational changes in the structure and that such changes are important and kinetically accessible in the course of the FEP simulation protocol.

One situation where one might imagine that the initial guess would present difficulties is in the case of additive mutations (i.e., the final residue is larger in size than the initial one). The great majority of mutations studied in this paper do not fall into this category, for the simple reason that alanine is the second smallest of the amino acids. When a larger residue is mutated to alanine, the structure is able to collapse around the space that is created when given sufficient time (doing so for Trp mutations understandably takes longer than for smaller residues). However, a Gly-to-Ala mutation could require substantial rearrangements of the structure.

One way to address this problem is to use the endpoint structure corresponding to the larger of the two residues in the alchemical pair as the initial guess. A structure of the Ala mutant can be generated using the loop and side-chain prediction methods in the PLOP program [39], [40]. We have used this approach to generate predicted Ala structures for all of the cases where the wild-type residue is a gly, and then, we run FEP simulations in which the Ala structure is employed as the initial guess. The results and comparison to the default protocol and experiment for individual cases can be found in the summary table (Table S5) in the Supplementary Data. For all cases but one, the errors were similar in magnitude (and consistent with the overall RMSE of the data set) for both starting points, suggesting that for these cases, the barriers to interconversion in the FEP protocol are unproblematic. However, for the Gly 54 residue on the PG04 antibody, a significant difference was observed, with the Ala starting point yielding results that are much closer to experiment.

Analysis of the Gly and Ala endpoint structures for this case provides insight into the nature of the problem. The PG04 antibody heavy chain contains two neighboring arginines, residues R71 and R73, whose interactions with gp120 can change under mutations on the CDR H2 loop. In the wild-type antibody, R71 forms an extremely stable salt bridge with an aspartic acid (D201 in our homology model, D368 in the 3SE9 crystal structure template used to build the RSC3 homology model) on the gp120, and R73 forms a stable contact with another residue on the antibody heavy chain. This salt bridge was found to be persistent in all other FEP trials of VRCPG-04 mutations and also across multiple independent MD simulations, long MD simulations of the crystal structure and homology model complex, and in PLOP predictions of the wild type CDR H2 loop with GLY at this position. In contrast, in the Ala structure, the R71 side chain is predicted to shift away from the D201 side chain, with R73 moved into contact with D201.

**Fig. 5:**
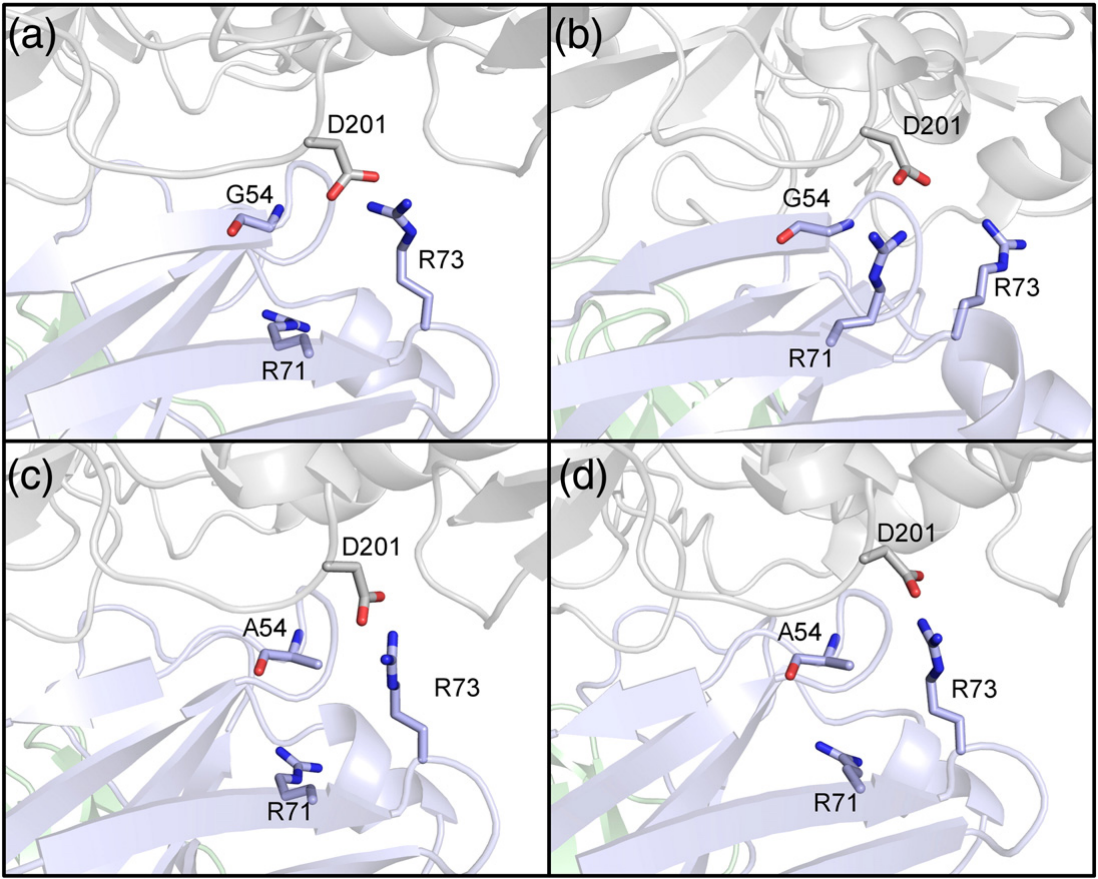
First and last frames of the (a to b) wild-type and (c to d) mutant phases for the VRC-PG04 G54 mutation using the PLOP-predicted starting structure. (b) The wild-type phase regains the contact with the gp120 residue shown, which is found for the crystal structure-based homology model and wild-type loop prediction. (c and d) The mutant phase system maintains the contact between R73 and the gp120 residue because the contact involving R71 is now prevented by the alanine side chain.

However, using the prediction for the mutant as the starting configuration for the FEP/REST run, we found that the mutant system stabilizes into a configuration where R73 replaces R71 in the contact with the gp120, approximately canceling the effect of breaking the contact with R71 (see Fig. 5). The barrier to the R73 crossing into this configuration is sufficiently large that a 100-ns trial is not sufficient to observe it, and it is doubtful that it will be accessible to any tractable simulation length. The large number of atoms involved in the barrier likely also makes it inaccessible to inclusion in the REST region. These results suggest that in prospective predictions going forward that involve larger additive mutations, applying the PLOP protocol to predict the conformation of the mutant state will be a useful adjunct in achieving robust, accurate relative binding affinity prediction. The net RMSE of the four CDR H2 loop cases was reduced from 1.78 kcal/mol using the default protocol to 0.89 kcal/mol. The remaining glycine-to-alanine cases on the CDR H3 and CDR L1 loops of VRCPG-04 mutations are identified as moderate or strong glycan contacts. Loop predictions for these cases were performed, with the glycan fragment present and included in the final protocol, and these are the values reported for the cases in Table 3. The difference from the result using the crystal structure loop was negligible in these cases.

### Summary of suggested protocol improvements for FEP calculations of the effect of mutation at a protein–protein interface

From the results discussed above, we can propose a protocol for running FEP calculations to predict the effects of protein mutations on protein–protein binding affinities. One study cannot completely define or validate a general protocol; it will require investigation of many more data sets to establish a prescription that can be viewed as fully reliable at high level of accuracy. Nevertheless, we have identified a number of key problems specific to protein–protein FEP (as opposed to small-molecule calculations) and developed solutions, which perform well overall for the calculations run to date. We do expect that our protocol will prove useful in predicting antibody/gp120 interactions, based on the size of the data set that we have considered, and the quality of the results.

The modifications we propose for protein–protein FEP calculations, as compared to protein–small-molecule calculations, can be summarized as follows:

(1) In small-molecule FEP, the great majority of calculations are reasonably well converged with 10 nsec of simulation time. In contrast, a subset of the cases above required simulation times of 100 nsec. This is clearly the case for tryptophan mutations and for residues in contact with glycans. These systems appear to require longer relaxation times, as is observed in trajectories of both the bound complex and (in at least one case) the antibody in solution. All such cases in the present data set have been run for 100 nsec, and the results are qualitatively superior to the 10-nsec runs. In future work, it may be the case that other types of mutations require longer running times; this is likely to be manifested in the lack of convergence at the 10-nsec simulation mark. Much more extensive exploration will be required to provide a complete, robust prescription as to when extended running times are required. One could simply run all calculations for 100 nsec (or longer); however, this necessitates the use of considerable additional GPU time. As GPU time becomes less expensive, it may make sense to adopt routine longer running times as a default protocol. At present, our specific recommendation is to run tryptophan mutations and glycan-involved mutations to 100 nsec and to carefully examine default 10-nsec runs for other calculations for signs of poor convergence.
(2) We have shown that continuum solvent-based loop and side-chain prediction can be helpful in improving the convergence of the FEP simulations when mutating a smaller residue (in the present cases, this is always a Gly residue) to a larger residue (in the present paper, it is always an Ala). The specific, recommended protocol is to carry out loop/side-chain prediction when the larger residue is the target residue and to use the predicted structure as the initial guess in the FEP simulations. In prospective design applications aimed at increasing potency, there will be many changes other than Gly to Ala that fall into this category; indeed, many, if not most, favorable mutations are likely to be additive. Comparing more extensive results of this type with the experiment will provide rigorous testing of this component of the recommended protocol in future work.

### Summary of results using recommended protocol

**Fig. 6:**
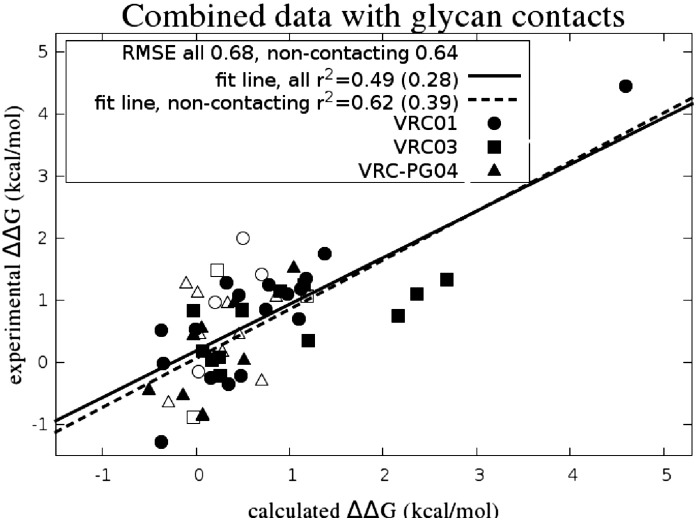
Experimental *versus* FEP/REST relative binding affinity values for alanine scan cases showing the combined data set from VRC01 (circles), VRC03 (squares), and VRC-PG04 (triangles). Correlation values with the largest experimental ΔΔG value excluded are given in parentheses.

Figure 6 presents the combined data for all three antibodies compared to the experiment. Table 4 summarizes separately the RMSE and correlation results for all three bNAbs and for the full data set with and without glycan contacts. VRC03 contained the largest computed outliers as compared to the experiment in the set, which may have been the result of the particularly strong chain of contacts between a glycan and the antibody light and heavy chain. This may have had some effect on other residues on the heavy chain not in a direct sequence of contact. The RMSE of the experimental results between re-measurements of the same system is estimated to be around 0.45 kcal/mol for a typical case (see the Supplementary Data Table S2 for details). The overall uncertainty range in our predictions with this protocol is comparable to the experimental levels, as is confirmed by the analysis of multiple independent FEP simulations, which gives an error estimate (full width) of about 0.51 kcal/mol (see Supplementary Data Table S7 for details). With the random error in the computed values and measurements both roughly 0.5 kcal/mol, the overall RMSE due to both theory and experiment would be expected to be around 0.7 kcal/mol, which is roughly what is found in the data presented here. While individual cases likely still exhibit systematic errors associated with the sampling or protein force field, the magnitude of the overall RMSE for the three data sets, 0.70 kcal/mol, suggests that it will be increasingly difficult to distinguish experimental from computational error unless the experimental error can be reduced.

### Insights from FEP trajectories

Analysis of trajectories generated from the FEP/REST alanine scan data can also be used to gain insights into the underlying reasons for the observed experimental and predicted changes in binding affinity. Here, we focus on three notable cases: W100B and G54 on VRC01, and F100D on VRC03. These cases are, respectively, the most unfavorable mutation experimentally, most favorable mutation experimentally, and an example of a case where the glycan affects a mutation indirectly through its direct interaction with another antibody residue, F91, on the light chain.

**Fig. 7:**
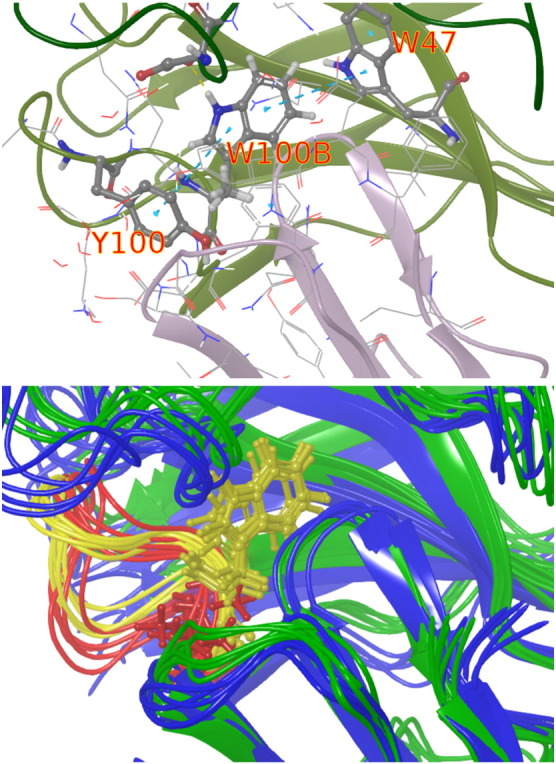
Top: Loss of the hydrogen bond of W100B (center) with gp120 residue A141 (dashed yellow line) and the stabilizing network of pi–pi stacking interactions (dashed light blue lines) result in the large unfavorable effect upon mutation to alanine. Bottom: Five frames from the end of the wild-type end (green) and five frames from the end of the mutant end trajectory (blue) with light chain backbones aligned to the first wild-type frame are shown with the H3 loops highlighted in yellow for the wild-type frames and red for the blue frames.

The first case, W100B, was the most unfavorable mutation in the alanine scan cases. Its indole nitrogen formed a highly stable hydrogen bond with a gp120 side chain (glu 204 in the RSC3 homology model) and also formed very stable pi-stacking interactions with two adjacent residues (Y100 and W47) that appear important in stabilizing the overall binding mode of VRC01 with gp120 (see the top panel of Fig. 7). The latter effectively anchored the long flexible CDR H3 loop to the sheet that W47 is on. In the bottom panel of Fig. 7, several frames from the wild type and mutant replicas are shown together, aligned to the backbone of the first wild-type frame. With W100B present, W100B (and Y100, not shown) is aligned well with the light chain, constraining the CDR H3 loop configuration. In the mutant end trajectory frames, in contrast, the much smaller alanine is essentially uncorrelated with the alignment of the light chain backbone, and the CDR H3 loop is able to assume a more favorable backbone configuration, explaining at least a part of the slow relaxation. The mutant trajectory frames also show a noticeable shift in the overall alignment between the heavy and light chains and the bNAb and the gp120. In addition to explaining the high unfavorability of the W100B to ALA mutation, this also explains why W47, which appears to have no direct contacts with the gp120, gave an unfavorable result upon mutation to ALA. The very stable contact with W100B also likely explains why Y100 on VRC01 showed little sensitivity to the presence of the glycan fragment NAG776, despite being in direct contact with it, as it could not shift into the space of the missing glycan when no fragment was included, due to the interaction with W100B. From the standpoint of future binding affinity optimization efforts, W100B and the residues contacting it are likely poor candidates for modification, as there is a large cost to destabilizing this network of interactions.

**Fig. 8:**
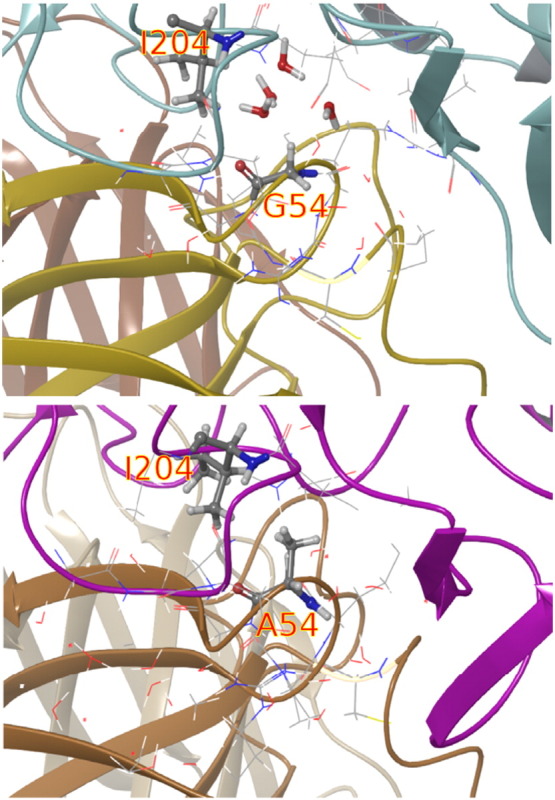
The mutation of G54 (top) to A54 on the heavy chain results in an improved hydrophobic contact with I204 on the gp120, resulting in the favorable change in binding affinity observed for this mutation.

G54 on the heavy chain of VRC01 was the most favorable mutation experimentally, and it can be seen from the trajectories that this is likely because the inserted alanine formed a favorable hydrophobic interaction with an isoleucine (I204 in the RSC3 homology model) on the gp120, whereas the glycine left a gap large enough for water to occupy (see Fig. 8). In the FEP simulations starting from the model structure (with the re-predicted mutant CDR H2 loop, as per the protocol outlined above), there were initially no waters present in the region between G54 and I204. The degree of water penetration differed with initial velocity seeds, resulting in variance somewhat higher than average in the FEP results, suggesting that future refinements to the protocol to better place solvent initially may help attain better convergence in certain cases.

The observed geometry in the trajectory frames also suggests the possibility that some further optimization of this hydrophobic contact might be possible by substituting valine or leucine for G54 instead of alanine. FEP/REST applied to mutations to valine predicts ΔΔ*G* of − 1.90, as its greater size is better able to fill the space between the CDR H2 loop and I204 on the gp120. It does not appear that further mutations to larger hydrophobic side chains will be able to further improve this, as mutation to leucine already requires a reorientation of side chains to avoid a clash. FEP/REST predicts that leucine performs comparably by assuming a different orientation from alanine and valine (ΔΔ*G* = − 1.79), but it is likely that larger residues will degrade this by destabilizing the nearby salt bridge between R71 on the bNAb heavy chain and D201 on the gp120 model. Thus, analysis of the alanine scan trajectories can potentially lead to information useful for design of hydrophobic optimizations.

**Fig. 9:**
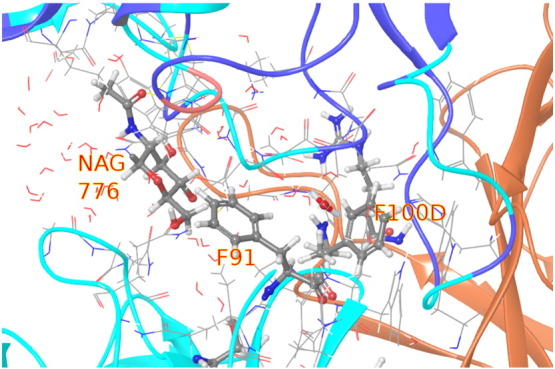
The hydrophobic contact between F100D and F91 does not form with the glycan fragment NAG776 included, leading to the significantly less unfavorable results consistent with experiment.

Finally, we consider an illustrative case that is sensitive to the presence of the glycan fragment and that shows how the sequences of aromatic side chains can propagate interactions with the glycan fragments not in direct contact with the residue to be mutated. F100D on the heavy chain of VRC03 formed a strong hydrophobic contact with the nearby residue F91 on the light chain; however, the presence of NAG776 in the model disrupted this contact, pushing away the F91 side chain far enough to allow water penetration between the two phenyl side chains (see Fig. 9). As a result of this, FEP without the glycan fragment NAG776 predicted these mutations as substantially more unfavorable than they are. Although the lack of the full glycan structure may slightly underestimate the unfavorability of the F91–glycan interaction, the results of FEP on the system including NAG776 are able to bring both to within 1 kcal/mol of the experimental result.

### Comparison with empirical method

**Fig. 10:**
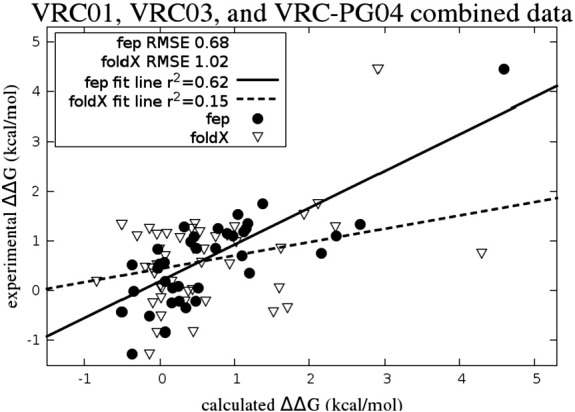
Combined set of mutations for VRC01, VRC03, and VRC-PG04 (filled circles) with FEP/REST compared with the results of the empirical foldX program (open squares).

For comparison, we show the results of FEP/REST in comparison to foldX [31], a popular empirical method of predicting alanine scan mutation values, which takes a negligible amount of computing time compared to the FEP/REST simulation time (less than 5 min per structure on a single processor *versus* ~ 12–72 h on 4 GPU cards for FEP/REST simulations). As foldX has no ability to treat the glycan fragment, we limit the comparison here to the 38 cases identified as insignificantly glycan-contacting. The results of FEP/REST over the full set showed a substantially lower RMSE (0.64 *versus* 1.02 kcal/mol) and a substantially better correlation (fit line *r*^2^ of 0.62 *versus* 0.15) than the results of foldX on the same set. The foldX RMSE of 1.02 kcal/mol is only slightly lower than the null hypothesis result of 1.14 kcal/mol obtained by taking all the calculated results to be 0. This favorable comparison holds up even with the removal of the largest experimental ΔΔ*G* value point from the set (RMSE 0.64 *versus* 1.00 for foldX; *r*^2^ = 0.39 *versus* *r*^2^ = 0.05 for foldX). Figure 10 shows the combined data from the 44 cases using FEP/REST together with the results from foldX for comparison. The foldX correlation coefficient is sufficiently small as to render the results of little utility in making actionable predictions in prioritizing mutations. The RMSE of foldX is respectable primarily because most of the predictions are close to zero free energy change, as are many of the experiments.

## Discussion

The experimental data presented in this study reveal that a subset of contact residues have greater impact on binding affinity, suggesting that these contacts account for the high binding affinity observed between VRC01-like antibodies and HIV-1 gp120. There are common locations across all three antibodies, which result in ΔΔ*G* values in excess of 1kal/mol when mutated to alanine, such as W47, W50, and R71. Another interesting result lies in the N terminus of all three antibodies' light chains. Alanine mutation of N-terminal contacting residues resulted in ΔΔ*G* lower than − 0.5 kcal/mol, suggesting that optimization in the area could improve antibody potency. Even though there are a number of commonalities among the three antibodies, their energy landscapes differ in a number of ways. VRC01 G54 mutant had a ΔΔ*G* of − 1.2 kcal/mol; however, in VRC03, similar contact G55 mutant had a ΔΔ*G* of 0.8 kcal/mol. In another example, in VRC-PG04, G100A had ΔΔ*G* of − 0.6 kcal/mol in contrast to VRC01 N100A mutants with a ΔΔ*G* of 1.1 kcal/mol.

The results here demonstrate that FEP/REST can be successfully applied across a large number of mutations in an antibody–antigen complex to give meaningful predictions of relative binding affinity. To our knowledge, this is the one of the first large-scale applications of FEP to any antibody–antigen complex, let alone one with the significant complications encountered here. The difficulties associated with homology modeling, glycosylation effects, relaxation times, and loop-level sampling of additive mutations appear to be effectively addressable with the current version of the FEP/REST software and the computational resources available. The level of accuracy presented here appears to be sufficient for screening prospective mutations for effectiveness in increasing binding affinity with a useful rate of success, although this will need to be validated by actual prospective calculations.

The current data set has provided a broad exploration of the issues that complicate the application of FEP/REST to protein complexes. While we have been able to demonstrate that long simulation times are required for some residue types and that even small additive mutations can result in large structural rearrangements necessitating the use of loop and side-chain prediction, more test sets are needed to generalize and refine these protocols.

There remain some outstanding sampling issues for a small handful of cases in the set. In particular, in one case on the H2 loop of VRC-PG04, T53 shows much more sensitivity to small changes in initial conditions than most of the other cases. This case is adjacent to the G54 case discussed in detail previously, and the difficulties may be of similar origin. An additional issue is that the case cited above is experimentally determined to result in a more favorable binding affinity, despite the reduction in the size of the residue from threonine to alanine. In general, one would expect that most such cases must arise because of favorable, relatively complex rearrangements in the surrounding residues at the interface, and capturing such rearrangements is a larger sampling problem than capturing effects more localized around the target residue. From our current results, longer simulation times on the order of 100 nsec appear unable to resolve these cases. More extensive conformational sampling might be able to address such cases with a higher degree of effectiveness. It is also possible, however, that experimental noise is a significant contributor to the disagreement between theory and experiment. Hence, a more intensive investigation of all aspects of these cases is called for in future works.

As noted previously, mutations in which the net charge of the system is changed present significantly greater difficulties for FEP simulations due to periodic boundary condition artifacts, as has been investigated by Parameswaran *et al.* and Rocklin *et al.* [27], [28], Lin and coworkers [41], and Reif and Oostenbrink [42]. These authors have suggested a posteriori corrections using Poisson–Boltzmann (PB) electrostatics to ameliorate these problems, a technique that appears to be promising. However, there are other issues, when the net charge of the system is changed by a mutation, which are not addressed by PB corrections, for example, the possibility of large structural rearrangements and the potential for changes in p*K*_*a*_ between wild-type and mutant systems. We are in the midst investigating the methodologies for correcting for boundary condition artifacts using PB corrections in conjunction with other strategies such as running the simulations with the experimental concentration of explicit ions and simultaneous creation/annihilation of counterions to keep the system neutral during the course of FEP simulations. The problems due to large-scale rearrangements and the changes of protonation state upon mutation pose fundamental issues in improving the sampling of the simulations to address major changes in loop conformations and the use of techniques such as constant pH simulation to handle protonation state changes. Finally, the accuracy of the potential energy function in cases where there is a change in charge needs to be calibrated; however, this can only be attempted when the key sampling issues have been addressed. A solution to these challenges is essential if FEP is to be used effectively in facilitating antibody design and optimization, as the change of a charged residue to a neutral or oppositely charged residue (or change of a neutral residue to a charged residue) will often be a useful modification.

## Models and Methods

### Experimental alanine scans

We mutated each contact of VRC01, VRC03, and VRC-PG04 to alanine, expressed the altered Fab antibody fragment, measured their affinities for HIV-1 gp120 using an Octet biosensor, and calculated the changes of Gibbs free energy (Δ*G*). Alteration of VRC01 glycine 54 to alanine enhanced affinities in some cases, suggesting that a hydrophobic residue at this position might lead to enhanced antibody potency. Characterizations of the interacting energy landscape between VRC01-like antibodies and HIV-1 gp120 thus provide a rational basis for germline origin and suggest ways to enhance potency.

VRC01, VRC03, and VRC-PG04 alanine mutants were generated by substituting individual antibody amino acids at the Ab-gp120 interface to alanine. Only contacting amino acids with buried surface area greater than 5 Å^2^ were chosen for the alteration (contacting residues defined in Zhou et al. [11]). HIV-1 gp120 core protein RSC3 was used for all binding experiments [10]. RSC3 protein and antibodies were produced in 293 FreeStyle cells and purified with a protein A immobilized 17b antibody affinity column and a protein A column, respectively.

A fortéBio Octet Red384 instrument was used to measure the binding kinetics of wild-type and alanine mutant antibodies to gp120. All assays were performed with agitation set to 1000 rpm in Kinetics Buffer (ForteBio). The final volume for all solution was 50 μl/well. Assays were performed at 30 °C in tilted black 384-well plates (Geiger Bio-One). Anti-Human Fc sensor tips (ForteBio) were used to capture Fabs for 300 s. Biosensor tips were then equilibrated for 90 s in Kinetics Buffer prior to measuring the association with RSC3 proteins in solution for 300 s. Sensor tips were then allowed to dissociate for 300 s. Parallel correction to subtract the systematic baseline drift was carried out by subtracting the measurements recorded for a loaded sensor incubated in Kinetics Buffer. Data analysis and curve fitting were carried out using Octet software, version 8.0. Experimental data were fitted with the binding equations describing a 1:1 interaction. Global and local analyses of the data sets assuming reversible binding (full dissociation) were carried out using nonlinear least-squares fitting, allowing a single set of binding parameters to be obtained simultaneously for all of the concentrations used in each experiment.

Binding free energy for each alanine mutant was calculated using the formula Δ*G* = *RT*ln(*K*_D_), where *R* is the ideal gas constant, *T* is the absolute temperature, and *K*_D_ is the dissociation constant (*K*_off_/*K*_on_). The change in the binding free energy between the alanine mutant and respective wild-type antibody (ΔΔ*G*) was calculated by subtracting Δ*G*_wt_ from Δ*G*_mutant_ with positive ΔΔ*G* signifying the decrease in affinity of the antibody to gp120 as a result of alanine substitution. Values for all mutations performed as part of this set are reported in Table S1.

### Homology model building methods for gp120/RSC3 complexes

For each of the three wild-type complexes, a model for the complex with the RSC3 gp120 was built using an available crystal structure (PDB structure 3NGB for VRC01, PDB structure 3SE8 for VRC03, and PDB structure 3SE9 for VRC-PG04). The sequence alignment between RSC3 and the gp120 strain in the template crystal structure was optimized using ClustalW [43], yielding a sequence identity of 50%, a sequence similarity of 63%, and gaps of 11%. The models were built using a knowledge-based approach (described in more detail in Ref. [44], where it was applied to the prediction of loops in homology models of antibodies), whereby insertions and deletions in the sequence alignment, and any missing backbone coordinates from the template itself, were reconstructed using a library of loop fragments of similar length from other known PDB structures. These candidate loop fragments were then filtered according to their stem geometry to discard those that cannot form reasonable connections with the existing model, and the surviving fragments were then positioned in the model by superposition of the attachment residues. Loops, which clash with the protein, or with each other, were discarded, and the surviving loops were then ranked by sequence similarity using BLOSSUM62 [45], with the highest sequence similarity loop being chosen.

The side-chain conformations of residues, which were conserved in the sequence alignment, were retained, while the conformations of all other side chains (including all residues involved in the loop building described above) were iteratively sampled using a coarse library of rotamers derived from known PDB structures until no clashes remained [44]. The coordinates of all atoms not derived directly from the template itself were then minimized, producing the final model. The antibodies were not present in the RSC3 model-building process; however, differences in the binding interface between the template structure taken from complexes with the three different antibodies are retained in the three different RSC3 models.

The antibody from the crystal structure was then aligned with this homology model. First, the homology model of the gp120 protein was aligned with the gp120 protein from the crystal structure bound to the antibody in such a way that the crystal structure antibody is moved by the same displacement and rotation. The antibody from the crystal structure was then merged with the homology model of the RSC3 gp120 sequence, using the relative orientation generated by aligning the gp120 proteins. Several rounds of side-chain optimization on the RSC3 gp120 protein in complex with the antibody were then performed using the Prime program [46], [47]. The regions of the antibody chains that are distant from the binding region were truncated to reduce the system size, and the resulting structure was prepared for MD simulations by adding hydrogens corresponding to physiological pH. The resulting set encompasses the mutations of 20 residues from VRC01, 11 from VRC03, and 15 from VRC-PG04, from their wild-type identity to alanine, across the three antibodies considered.

The net difference between the common parts of the gp120 protein in the crystal structure from the gp120 homology model is quite small; the heavy atom RMSDs for the common parts of the template and RSC3 sequence are 0.66 Å for VRC01 (0.38 Å for only backbone atoms), 0.37 Å for VRC03 (0.38 Å for only backbone atoms), and 0.45 Å for VRC-PG04 (0.40 Å for backbone atoms). No residues whose backbones had to be predicted because of insertions or deletions from the template structure are within 5 Å of any residue on the antibody, and only a single residue backbone insertion point is within 10 Å of the antibody. The backbone structure in the interface region is nearly identical to the template, save for one loop in the template that does not occur in the RSC3 model. A figure comparing the homology model and the 3NGB crystal structure is available in the Supplementary Data (Fig. S1).

Each antibody-homology model complex was further validated with a 100-ns MD simulation to verify that the binding properties with the antibody using the homology model were stable under thermal conditions. Comparing simulations using the template crystal structures shows that the identified binding regions show comparable stabilization in both crystal structures and homology models, with backbone RMSDs from the input structure in both stabilizing below 2.0 Å. Details may be found in the Supplementary Data (Fig. S3). We conclude from this that the homology model provides a reasonable description of the binding interface.

Given the close homology in the CD4-binding region between the gp120 variant used in the experiments and in the crystal structures of 3NGB, 3SE8, and 3SE9, it is a plausible hypothesis that the glycans in the experimental system occupy similar positions in the experimental gp120/antibody complex as in the crystal structures. As noted previously, only one of the glycan fragments, NAG776, is close enough to any of the sites of mutation to induce a large direct effect on the change in binding free energy upon mutation.

**Fig. 11:**
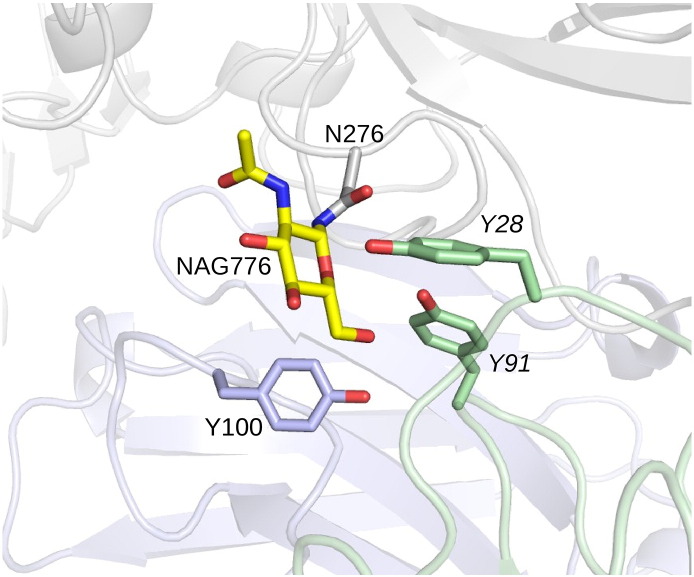
The glycan fragment on gp120 residue N276 captured in the 3NGB crystal structure, N776, is shown with the three cases identified as suspected glycan contacts (Y104, Y91, and Y104).

Figure 11 shows the glycan fragment NAG776 in the 3NGB structure, which is in clear contact with the antibody-binding region. Based on the hypothesis that the effects of NAG776 can be largely captured by including the part that has been observed in the crystal structures, we performed FEP/REST on the 17 cases identified as potential direct or indirect glycan contacts from the crystal structure. Short MD simulations were performed to determine likely glycan contacts. Direct contact score was defined by a simple interatomic contact count, scaled with a sigmoidal cutoff around 4 Å, and averaged over simulation frames. Indirect contacts were defined by scoring contacts of aromatic side-chain residues (tryptophan, tyrosine, and phenylalanine) with direct contacts and aromatic side-chain contacts of direct contacts and with a higher contact score threshold. Details of the scoring parameters used are provided in the Supplementary Data. While in some cases, the fragment included may not capture the full set of interactions with the glycan, especially for residues on the light chain away from the heavy light chain interface, for many cases, the inclusion of the Nag776 fragment gives predictions in significant agreement with the experimental results.

### FEP MD simulations

#### Default small molecular protocol (10-nsec runs)

All simulations were performed using the Desmond MD program [48], [49], [50]. Input structures are solvated with a 5-Å buffer of simple point charge (spc) water in a rectangular box. For each case, a short relaxation phase, with a combination of minimization and restrained MD phases, was performed to equilibrate the system. Each equilibrated structure was then input into a 10-ns FEP/REST simulation to calculate the change in Gibbs free energy, Δ*G*, with the difference between the bound and unbound simulations giving the relative binding affinity (ΔΔ*G*). This protocol uses 12 lambda windows in the FEP/REST schedule run for 10 ns with the side chain of the residue being mutated included in the REST hot region. The FEP/REST phase was performed in the Isothermal-isobaric ensemble with a Berendsen thermostat and barostat. The equations of motion were integrated using a reversible reference system propagator algorithm (RESPA) scheme with an inner time step of 2.0 fs and an outer time step of 6.0 fs.

#### Modified simulation protocols

For TRP cases, and cases where the glycan fragment is included in the model gp120–bNAb complex, simulation times for the FEP/REST phase of the simulation are extended from 10 ns to 100 ns. For glycine-to-alanine mutations, the antibody loop containing the glycine residue is re-predicted, along with surrounding side-chain conformations and with the alanine replacing the glycine. This structure with the re-predicted loop on the antibody is then used as the input for the full simulation protocol, with the same equilibration cycle and 10-ns (100 ns for glycan contacts) FEP/REST simulation cycle. For glycan contacts, the loop prediction is performed with the glycan fragment present in the structure. All cases with the glycan fragment present use 100-ns FEP/REST phases. In addition, for any remaining 10-ns simulations, which show greater than 0.5 kcal/mol differences between the two independent trials of one simulation leg, three additional independent trials seeded with different, randomly generated initial velocities are performed and included in the average.

## Conclusions

We have shown here a proof of concept that FEP/REST can be used to predict the relative binding affinities of a large set of alanine scan data. Several protocol refinements and modeling improvements have been put forward, which reduce the overall error in the data set to a level that is comparable to the estimated experimental uncertainty. As noted above, the present protocol is incomplete in that we have not yet demonstrated the ability to treat mutations, in which the net charge on the protein is changed. The use of PB calculations to correct for periodic boundary condition effects, combined with the inclusion of appropriate ionic strength, is a promising approach to this problem and can readily be tested using the data sets discussed here. Work along these lines is currently in progress.

While there is reason to be optimistic that the protocols described here could be profitably applied to predict the effects of mutation for a wide range of protein–protein interactions, new problems may arise for different systems. Hence, application of the present methodology across a diverse set of protein–protein complexes is essential for assessment of broad applicability. We plan to report the results for such data sets in subsequent publications.

## Figures and Tables

**Table 1:** RMSE and correlation coefficients for the default protocol

	RMSE (kcal/mol)	Correlation coefficient	Worst outlier
VRC01	0.64	0.71	L_Y28: 1.61 kcal/mol
VRC03	1.72	0.23	L_F91: 2.70 kcal/mol
VRCPG-04	1.18	0.12	H_G54: 3.19 kcal/mol
combined	1.19	0.28	H_G54: 3.19 kcal/mol

**Table 2:** Summary of the five TRP-> ALA cases in the data set, which are not strong or moderate glycan contact cases

Case	Experimental ΔΔ*G*	10-ns FEP/REST ΔΔ*G*	100-ns FEP/REST ΔΔ*G*	Change in absolute error
VRC01 W100B	4.45	4.39	4.59	0.08
VRC01 W50	1.28	0.61	0.33	0.28
VRC01 W47	1.19	1.71	1.12	− 0.45
VRC03 W50	1.33	4.36	2.68	− 1.68
VRC03 W54	0.76	3.46	2.16	− 1.30

In three of the five cases, significant improvement is obtained from increased simulation time. All values are free energies in units of kcal/mol.

**Table 3:** Information on glycan-contacting cases

Glycan score	bNAb	Chain	Mutation	Experiment	10 ns without glycan fragment	10 ns with glycan fragment	100 ns with glycan fragment
9.96	VRC01	heavy	Y100	1.42	0.69	1.03	0.7
10.29	VRC01	light	Y91	2.01	1.26	1.67	1.02
11.76	VRC01	light	Y28	0.97	− 0.63	0.36	0.28
4.31	VRC01	light	S30	− 0.15	0.08	0.57	0.03
23.01	VRC03	heavy	W47	1.48	3.96	3.92	0.23
19.69	VRC03	heavy	F100D	1.07	4.48	4.1	1.63
27.69	VRC03	light	F91	− 0.87	1.84	0.26	0.38
69.35	VRC03	light	F97	0.83	0.79	1.98	0.51
28.68	VRC-PG04	heavy	W50	1.28	2.78	− 0.71	− 0.11
21.49	VRC-PG04	heavy	W47	1.07	1.18	− 0.35	0.86
20.28	VRC-PG04	heavy	G100A	− 0.63	− 0.17	0.14	− 0.37
13.78	VRC-PG04	heavy	Y98	0.47	1.37	− 0.25	− 0.02
4.69	VRC-PG04	heavy	G100	− 0.27	1.12	0.76	0.7
21.48	VRC-PG04	heavy	G100C	0.19	0.07	− 0.11	0.28
23	VRC-PG04	light	Y30	1.13	− 0.16	0.23	− 0.05
26.13	VRC-PG04	light	L91	0.97	− 0.69	0.31	0.34
20.4	VRC-PG04	light	G31	0.47	− 0.14	− 0.03	0.04
26.5	VRC-PG04	light	F97	0.02	− 0.02	0.74	0.17
					RMSE 1.47	RMSE 1.27	RMSE 0.77

Use of the glycan-fragment containing model appears to require longer simulation times. The total RMSE for glycan-contacting cases is reduced from 1.47 kcal/mol without the glycan fragment to 0.77 kcal/mol with the glycan fragment present and 100-ns runs. The reduction in RMSE is much more modest (1.47 to 1.27 kcal/mol) using the glycan fragment-containing model with the default 10-ns simulation length.

**Table 4:** Summary of results for the improved protocol

	Without glycan-contacting cases		Including glycan-contacting cases	
Data set	RMSE	Correlation	RMSE	Correlation
VRC01	0.55	0.79	0.64	0.72
VRC03	0.78	0.41	0.78	0.27
VRCPG-04	0.58	0.49	0.65	0.22
All three antibodies	0.64	0.62	0.68	0.49

RMSE values are in kcal/mol.

## Appendix A Supplementary Data

Supplementary material Image 2

## References

[1] Stamatatos L., Morris L., Burton D.R., Mascola J.R. (2009). Neutralizing antibodies generated during natural HIV-1 infection: good news for an HIV-1 vaccine?. Nat. Med..

[2] Li Y., Migueles S.A., Welcher B., Svehla K., Phogat A., Louder M.K., Wu X., Shaw G.M., Connors M., Wyatt R.T., Mascola J.R. (2007). Broad HIV-1 neutralization mediated by CD4-binding site antibodies. Nat. Med..

[3] Kwong P.D., Mascola J.R. (2012). Human antibodies that neutralize HIV-1: identification, structures, and B cell ontogenies. Immunity.

[4] Wu X., Zhou T., Zhu J., Zhang B., Georgiev I., Wang C., Chen X., Longo N.S., Louder M., McKee K., O’Dell S., Perfetto S., Schmidt S.D., Shi W., Wu L., Yang Y., Yang Z.-Y., Yang Z., Zhang Z., Bonsignori M., Crump J.A., Kapiga S.H., Sam N.E., Haynes B.F., Simek M., Burton D.R., Koff W.C., Doria-Rose N.A., Connors M., Mullikin J.C., Nabel G.J., Roederer M., Shapiro L., Kwong P.D., Mascola J.R. (2011). Focused evolution of HIV-1 neutralizing antibodies revealed by structures and deep sequencing. Science.

[5] Wu X., Zhang Z., Schramm C.A., Joyce M.G., Do Kwon Y., Zhou T., Sheng Z., Zhang B., O'Dell S., McKee K., Georgiev I.S., Chuang G.-Y., Longo N.S., Lynch R.M., Saunders K.O., Soto C., Srivatsan S., Yang Y., Bailer R.T., Louder M.K., Mullikin J.C., Connors M., Kwong P.D., Mascola J.R., Shapiro L. (2015). Maturation and diversity of the VRC01-antibody lineage over 15 years of chronic HIV-1 infection. Cell.

[6] Balazs A.B., Chen J., Hong C.M., Rao D.S., Yang L., Baltimore D. (2011). Antibody-based protection against HIV infection by vectored immunoprophylaxis. Nature.

[7] Sather D.N., Armann J., Ching L.K., Mavrantoni A., Sellhorn G., Caldwell Z., Yu X., Wood B., Self S., Kalams S., Stamatatos L. (2009). Factors associated with the development of cross-reactive neutralizing antibodies during human immunodeficiency virus type 1 infection. J. Virol..

[8] Hraber P., Seaman M.S., Bailer R.T., Mascola J.R., Montefiori D.C., Korber B.T. (2014). Prevalence of broadly neutralizing antibody responses during chronic HIV-1 infection. AIDS.

[9] Ledgerwood J.E., Coates E.E., Yamshchikov G., Saunders J.G., Holman L., Enama M.E., Dezure A., Lynch R.M., Gordon I., Plummer S., Hendel C.S., Pegu A., Conan-Cibotti M., Sitar S., Bailer R.T., Narpala S., McDermott A., Louder M., O’Dell S., Mohan S., Pandey J.P., Schwartz R.M., Hu Z., Koup R.A., Capparelli E., Mascola J.R., Graham B.S., Mendoza F., Novik L., Zephir K., Whalen W., Larkin B., Vasilenko O., Berkowitz N., Wilson B., Pittman I., Schieber G., Decederfelt H., Starling J., Gilly J., Rao S., Kaltovich F., Renehan P., Kunchai M., Romano S., Menard K., Diep L., Anude C., Allen M. (2015). Safety, pharmacokinetics and neutralization of the broadly neutralizing HIV-1 human monoclonal antibody VRC01 in healthy adults. Clin. Exp. Immunol..

[10] Wu X., Yang Z.-Y., Li Y., Hogerkorp C.-M., Schief W.R., Seaman M.S., Zhou T., Schmidt S.D., Wu L., Xu L., Longo N.S., McKee K., O'Dell S., Louder M.K., Wycuff D.L., Feng Y., Nason M., Doria-Rose N., Connors M., Kwong P.D., Roederer M., Wyatt R.T., Nabel G.J., Mascola J.R. (2010). Rational design of envelope identifies broadly neutralizing human monoclonal antibodies to HIV-1. Science.

[11] Zhou T., Georgiev I., Wu X., Yang Z.-Y., Dai K., Finzi A., Do Kwon Y., Scheid J.F., Shi W., Xu L., Yang Y., Zhu J., Nussenzweig M.C., Sodroski J., Shapiro L., Nabel G.J., Mascola J.R., Kwong P.D. (2010). Structural basis for broad and potent neutralization of HIV-1 by antibody VRC01. Science.

[12] Zhou T., Lynch R.M., Chen L., Acharya P., Wu X., Doria-Rose N.A., Joyce M.G., Lingwood D., Soto C., Bailer R.T., Ernandes M.J., Kong R., Longo N.S., Louder M.K., McKee K., O’Dell S., Schmidt S.D., Tran L., Yang Z., Druz A., Luongo T.S., Moquin S., Srivatsan S., Yang Y., Zhang B., Zheng A., Pancera M., Kirys T., Georgiev I.S., Gindin T., Peng H.P., Yang A.S., Mullikin J.C., Gray M.D., Stamatatos L., Burton D.R., Koff W.C., Cohen M.S., Haynes B.F., Casazza J.P., Connors M., Corti D., Lanzavecchia A., Sattentau Q.J., Weiss R.A., West A.P., Bjorkman P.J., Scheid J.F., Nussenzweig M.C., Shapiro L., Mascola J.R., Kwong P.D. (2015). Structural repertoire of HIV-1-neutralizing antibodies targeting the CD4 supersite in 14 donors. Cell.

[13] Zhou T., Zhu J., Wu X., Moquin S., Zhang B., Acharya P., Georgiev I.S., Altae-Tran H.R., Chuang G.-Y., Joyce M.G., Do Kwon Y., Longo N.S., Louder M.K., Luongo T., McKee K., Schramm C.A., Skinner J., Yang Y., Yang Z., Zhang Z., Zheng A., Bonsignori M., Haynes B.F., Scheid J.F., Nussenzweig M.C., Simek M., Burton D.R., Koff W.C., Mullikin J.C., Connors M., Shapiro L., Nabel G.J., Mascola J.R., Kwong P.D. (2013). Multidonor analysis reveals structural elements, genetic determinants, and maturation pathway for HIV-1 neutralization by VRC01-class antibodies. Immunity.

[14] Diskin R., Scheid J.F., Marcovecchio P.M., West a.P., Klein F., Gao H., Gnanapragasam P.N.P., Abadir a., Seaman M.S., Nussenzweig M.C., Bjorkman P.J. (2011). Increasing the potency and breadth of an HIV antibody by using structure-based rational design. Science.

[15] Sok D., van Gils M.J., Pauthner M., Julien J.-P., Saye-Francisco K.L., Hsueh J., Briney B., Lee J.H., Le K.M., Lee P.S., Hua Y., Seaman M.S., Moore J.P., Ward A.B., Wilson I.a., Sanders R.W., Burton D.R., van Gils S.D. (2014). Recombinant HIV envelope trimer selects for quaternary-dependent antibodies targeting the trimer apex. Proc. Natl. Acad. Sci..

[16] Rudicell R.S., Kwon Y.D., Ko S.-Y., Pegu A., Louder M.K., Georgiev I.S., Wu X., Zhu J., Boyington J.C., Chen X., Shi W., Yang Z.-Y., Doria-Rose N.A., McKee K., O’Dell S., Schmidt S.D., Chuang G.-Y., Druz A., Soto C., Yang Y., Zhang B., Zhou T., Todd J.-P., Lloyd K.E., Eudailey J., Roberts K.E., Donald B.R., Bailer R.T., Ledgerwood J., Mullikin J.C., Shapiro L., Koup R.A., Graham B.S., Nason M.C., Connors M., Haynes B.F., Rao S.S., Roederer M., Kwong P.D., Mascola J.R., Nabel G.J. (2014). Enhanced potency of a broadly neutralizing HIV-1 antibody *in vitro* improves protection against lentiviral infection *in vivo*. J. Virol..

[17] Wang L., Berne B.J., Friesner R.A. (2012). On achieving high accuracy and reliability in the calculation of relative protein-ligand binding affinities. Proc. Natl. Acad. Sci. U. S. A..

[18] Wang L., Wu Y., Deng Y., Kim B., Pierce L., Krilov G., Lupyan D., Robinson S., Dahlgren M.K., Greenwood J., Romero D.L., Masse C., Knight J.L., Steinbrecher T., Beuming T., Damm W., Harder E., Sherman W., Brewer M., Wester R., Murcko M., Frye L., Farid R., Lin T., Mobley D.L., Jorgensen W.L., Berne B.J., Friesner R.A., Abel R. (2015). Accurate and reliable prediction of relative ligand binding potency in prospective drug discovery by way of a modern free-energy calculation protocol and force field. J. Am. Chem. Soc..

[19] Jorgensen W.L. (2009). Efficient drug lead discovery and optimization. Acc. Chem. Res..

[20] Gallicchio E., Levy R.M. (2011). Advances in all atom sampling methods for modeling protein–ligand binding affinities. Curr. Opin. Struct. Biol..

[21] Sambasivarao S.V. (2013). NIH public access. Curr. Opin. Struct. Biol..

[22] Wang L., Friesner R.A., Berne B.J. (2011). Replica exchange with solute scaling: a more efficient version of replica exchange with solute tempering (REST2). J. Phys. Chem. B.

[23] Wang L., Deng Y., Knight J.L., Wu Y., Kim B., Sherman W., Shelley J.C., Lin T., Abel R. (2013). Modeling local structural rearrangements using FEP/REST: application to relative binding affinity predictions of CDK2 inhibitors. J. Chem. Theory Comput..

[24] Park H., Jeon Y.H. (2011). Free energy perturbation approach for the rational engineering of the antibody for human hepatitis B virus. J. Mol. Graph. Model..

[25] Xia Z., Huynh T., Kang S., Zhou R. (2012). Free-energy simulations reveal that both hydrophobic and polar interactions are important for influenza hemagglutinin antibody binding. Biophys. J..

[26] Zhou R., Das P., Royyuru A.K. (2008). Single mutation induced H3N2 hemagglutinin antibody neutralization: a free energy perturbation study. J. Phys. Chem. B.

[27] Parameswaran S., Mobley D.L. (2014). Box size effects are negligible for solvation free energies of neutral solutes. J. Comput. Aided Mol. Des..

[28] Rocklin G.J., Mobley D.L., Dill K.A., Hünenberger P.H. (2013). Calculating the binding free energies of charged species based on explicit-solvent simulations employing lattice-sum methods: an accurate correction scheme for electrostatic finite-size effects. J. Chem. Phys..

[29] Go E.P., Herschhorn A., Gu C., Castillo-Menendez L., Zhang S., Mao Y., Chen H., Ding H., Wakefield J.K., Hua D., Liao H.-X., Kappes J.C., Sodroski J., Desaire H. (2015). Comparative analysis of the glycosylation profiles of membrane-anchored HIV-1 envelope glycoprotein trimers and soluble gp140. J. Virol..

[30] Pritchard L.K., Harvey D.J., Bonomelli C., Crispin M., Doores K.J. (2015). Cell- and protein-directed glycosylation of native cleaved HIV-1 envelope. J. Virol..

[31] Guerois R., Nielsen J.E., Serrano L. (2002). Predicting changes in the stability of proteins and protein complexes: a study of more than 1000 mutations. J. Mol. Biol..

[32] Li Y., Svehla K., Louder M.K., Wycuff D., Phogat S., Tang M., Migueles S.a., Wu X., Phogat A., Shaw G.M., Connors M., Hoxie J., Mascola J.R., Wyatt R. (2009). Analysis of neutralization specificities in polyclonal sera derived from human immunodeficiency virus type 1-infected individuals. J. Virol..

[33] Clades A., Stewart-jones G.B.E., Soto C., Lemmin T., Wong C., Mascola J.R., Kwong P.D. (2016). Trimeric HIV-1-Env structures define glycan shields from clades A, B, and G. Cell.

[34] Stewart-Jones G.B., Soto C., Lemmin T., Chuang G.Y., Druz A., Kong R. (2016). Trimeric HIV-1-Env Structures Define Glycan Shields from Clades A, B, and G. Cell.

[35] Gristick H.B., von Boehmer L., West A.P., Schamber M., Gazumyan A., Golijanin J. (2016). Natively glycosylated HIV-1 Env structure reveals new mode for antibody recognition of the CD4-binding site. Nat. Struct. Mol. Biol..

[36] Jardine J.G., Sok D., Julien J.P., Briney B., Sarkar A., Liang C.H. (2016). Minimally Mutated HIV-1 Broadly Neutralizing Antibodies to Guide Reductionist Vaccine Design. PLoS Pathog..

[37] Scharf L., Wang H., Gao H., Chen S., McDowall A.W., Bjorkman P.J. (2015). Broadly Neutralizing Antibody 8ANC195 Recognizes Closed and Open States of HIV-1 Env. Cell.

[38] Harder E., Damm W., Maple J., Wu C., Reboul M., Xiang J.Y., Wang L., Lupyan D., Dahlgren M.K., Knight J.L., Kaus J.W., Cerutti D.S., Krilov G., Jorgensen W.L., Abel R., Friesner R.A. (2015). OPLS3: a force field providing broad coverage of drug-like small molecules and proteins. J. Chem. Theory Comput..

[39] Friesner R.A., Abel R., Goldfeld D.A., Miller E.B., Murrett C.S. (2013). Computational methods for high resolution prediction and refinement of protein structures. Curr. Opin. Struct. Biol..

[40] Miller E.B., Murrett C.S., Zhu K., Zhao S., Goldfeld D.a., Bylund J.H., Friesner R.A. (2013). Prediction of long loops with embedded secondary structure using the protein local optimization program. J. Chem. Theory Comput..

[41] Lin Y., Aleksandrov A., Simonson T. (2014). An overview of electrostatic free energy computations for solutions and proteins. J. Chem. Theory Comput..

[42] Reif M.M., Oostenbrink C. (2014). Net charge changes in the calculation of relative ligand-binding free energies via classical atomistic molecular dynamics simulation. J. Comput. Chem..

[43] Thompson J.D., Higgins D.G., Gibson T.J. (1994). CLUSTAL W: improving the sensitivity of progressive multiple sequence alignment through sequence weighting, position-specific gap penalties and weight matrix choice. Nucleic Acids Res..

[44] Zhu K., Day T., Warshaviak D., Murrett C., Friesner R., Pearlman D. (2014). Antibody structure determination using a combination of homology modeling, energy-based refinement, and loop prediction. Proteins Struct. Funct. Bioinforma..

[45] Henikoff S., Henikoff J.G. (1992). Amino acid substitution matrices from protein blocks. Proc. Natl. Acad. Sci. U. S. A..

[46] Jacobson M.P., Pincus D.L., Rapp C.S., Day T.J.F., Honig B., Shaw D.E., Friesner R.A. (2004). A hierarchical approach to all-atom protein loop prediction. Proteins Struct. Funct. Bioinforma..

[47] Jacobson M.P., Friesner R.A., Xiang Z., Honig B. (2002). On the role of the crystal environment in determining protein side-chain conformations. J. Mol. Biol..

[48] SC '06: Proceedings of the 2006 ACM/IEEE Conference on Supercomputing;ACM, New York, NY, USA; 2006.

[49] Shivakumar D., Williams J., Wu Y., Damm W., Shelley J., Sherman W. (2010). Prediction of absolute solvation free energies using molecular dynamics free energy perturbation and the opls force field. J. Chem. Theory Comput..

[50] Guo Z., Mohanty U., Noehre J., Sawyer T.K., Sherman W., Krilov G. (2010). Probing the alpha-helical structural stability of stapled p53 peptides: molecular dynamics simulations and analysis. Chem. Biol. Drug Des..

