# Supplementary material for: Free Energy Perturbation Calculation of Relative Binding Free Energy between Broadly Neutralizing Antibodies and the gp120 Glycoprotein of HIV-1
Source: J Mol Biol. 2017 Apr 7;429(7):930–47. doi: 10.1016/j.jmb.2016.11.021 (PMC5383735; doi:10.1016/j.jmb.2016.11.021)
Supplement: Supplementary file 1 — Supplementary material [file mmc1.pdf]

Supplemental Information

Experimental Data

Average dissociation constant values and conversion to ΔΔG values

Table S1 lists the average value of the dissociation constant (K_D) over all independent trials performed for each. The first line gives K_D for the wild type system, and subsequent rows list the values obtained with the sequence with the single point mutation listed as well as the relative change in gibbs free energy of binding converted from the average dissociation constants. The latter values are used in all comparisons with the results from FEP in the main text. For cases where multiple trials were performed, the average K_D value is used.

	**K_D (nM)**	**ΔΔG (kcal/mol)**		**K_D (nM)**	**ΔΔG (kcal/mol)**		**K_D (nM)**	**ΔΔG (kcal/mol)**
VRC01	22	--	VRC03	63	--	VRC-PG04	25	--
Heavy Chain								
I30	21.9	-0.01	R30	59	-0.04	L34	64	0.57
T33	71.2	0.7	W47	730	1.48	W47	145	1.07
W47	161.5	1.19	W50	577	1.33	W50	208	1.53
W50	187.7	1.28	K52	399	1.11	V52B	6	-0.82
K52	61.93	0.62	L54	69	0.05	T53	6	-0.85
R53	14.3	-0.27	W55	223	0.76	G54	53	0.46
G54	2.64	-1.28	G56	114	0.35	V56	128	0.99
G55	180	1.26	V58	502	1.25	N57	313	1.53
V57	209.7	1.35	S59	426	1.15	G59	27	0.06
N58	413.5	1.76	Y60	261	0.86	P61	12	-0.43
Y59	54.73	0.54	R62	467	1.21	R64	287	1.48
R61	114.9	0.99	Q63	401	1.11	R71	2,140	2.69
P62	20.5	-0.05	Q65	157	0.55	R73	17	-0.22
Q64	12.5	-0.35	R72	5,090	2.65	D74	7	-0.23
M69	53.05	0.52	Q76	74	0.09	Y98	54	0.47
R71	337	1.64	P78	65	0.01	T99	12	-0.43
V73	134	1.08	P81	56	-0.08	G100	15	-0.27
Y74	15.75	-0.21	D110	33	-0.38	G100A	9	-0.63
D99	24.25	0.05	Y111	250	0.83	G100C	34	0.19
Y100	234	1.42	D114	42	-0.24	G100D	120	0.95
N100A	141	1.11	F115	371	1.07			
W100B	36350	4.45						

Light chain										
V3	6.09	-0.78		E1	25	-0.57		V3	11	-0.51
Q27	15	-0.24		Q27	44	-0.22		Y30	160	1.13
Y28	112.5	0.97		N30	87	0.2		G31	54	0.47
S30	17.3	-0.15		D49	261	0.86		L91	124	0.97
Y91	632.7	2.01		F90	15	-0.87		E96	50	0.42
E96	197	1.31		E91	778	1.51		F97	26	0.02
F97	91.55	0.85		F92	251	0.83				

Table S1: Experimentally determined antibody – HIV-1 gp120 binding kinetics for the wild type antibodies and alanine mutants.

Estimation of experimental error

To estimate the error in the relative free energies multiple independent K_D measurements for a large subset of cases are presented in table S2. The results indicate an average random variation of about 0.45 kcal/mol in the experimental ΔΔG values.

Case	Trial 1	Trial 2	Trial 3	Estimated full uncertainty width
VRC01 Heavy chain				
I30	-0.27	0.11	0.06	0.49
T33	0.84	0.52	--	0.54
W47	1.35	0.98	--	0.62
W50	1.20	1.31	1.33	0.17
K52	0.85	0.67	0.09	0.93
R53	-0.44	-0.47	-0.01	0.60
G54	-1.05	-1.66	--	1.01
G55	1.35	1.15	--	0.34
V57	1.58	1.30	1.03	0.65
N58	1.58	1.89	--	0.51
Y59	0.63	0.70	0.16	0.69
R61	0.76	1.19	0.92	0.51
P62	0.09	-0.39	0.04	0.62
M69	0.86	-0.32	--	1.97
R71	1.31	1.84	--	0.88
Y74	-0.10	-0.35	--	0.42
D99	0.29	-0.36	--	1.08
Y100	1.51	1.30	--	0.35
N100A	1.14	1.07	--	0.11
W100B	4.76	3.76	--	1.67
Light chain				

V3	-0.74	-0.65	-1.02	0.45
Q27	-0.44	-0.12	-0.20	0.39
Y28	0.99	0.95	--	0.06
Y91	2.21	1.67	2.04	0.64
E96	1.18	1.42	--	0.39
F97	1.04	0.57	--	0.79
VRC03 light chain				
R30	0.04	-0.14	--	0.29
W47	1.57	1.36	--	0.36
W50	1.40	1.26	--	0.23
K52	1.01	1.19	--	0.31
L54	-0.10	0.17	--	0.45
W55	0.88	0.60	--	0.47
G56	0.30	0.40	--	0.17
S59	1.09	1.20	--	0.19
Y60	0.83	0.88	--	0.08
R62	1.18	1.23	--	0.08
E1	-0.57	--	--	
Q27	-0.22	--	--	
N30	0.19	--	--	
D49	0.85	--	--	
F90	-0.87	--	--	
E91	1.51	--	--	
92	0.83	--	--	
L34	0.01	--	--	
W47	0.50	--	--	
W50	0.66	0.77	--	0.18
V52B	-1.38	--	--	
T53	-1.19	-1.75	--	0.93
G54	-0.11	--	--	
V56	0.43	--	--	
N57	0.79	1.10	--	0.51
G59	-0.50	--	--	
S60	-0.83	--	--	

P61	-0.99	--	--	
R64	0.83	0.99	--	0.27
R71	2.12	--	--	
R73	-0.80	-0.78	--	0.04
		Median error width		0.45

Table S2: Experimental re-measurement statistics used to estimate the experimental uncertainty stated in the main text. All values are in kcal/mol.

Homology Model

Comparison homology model to crystal structure for gp120 in complex with VRC01

Figure S1 shows the 3NGB crystal structure with the RSC3 homology model built using the gp120 from 3NGB as a template. Most variation in the backbone structure between the two occurs far from the binding region.

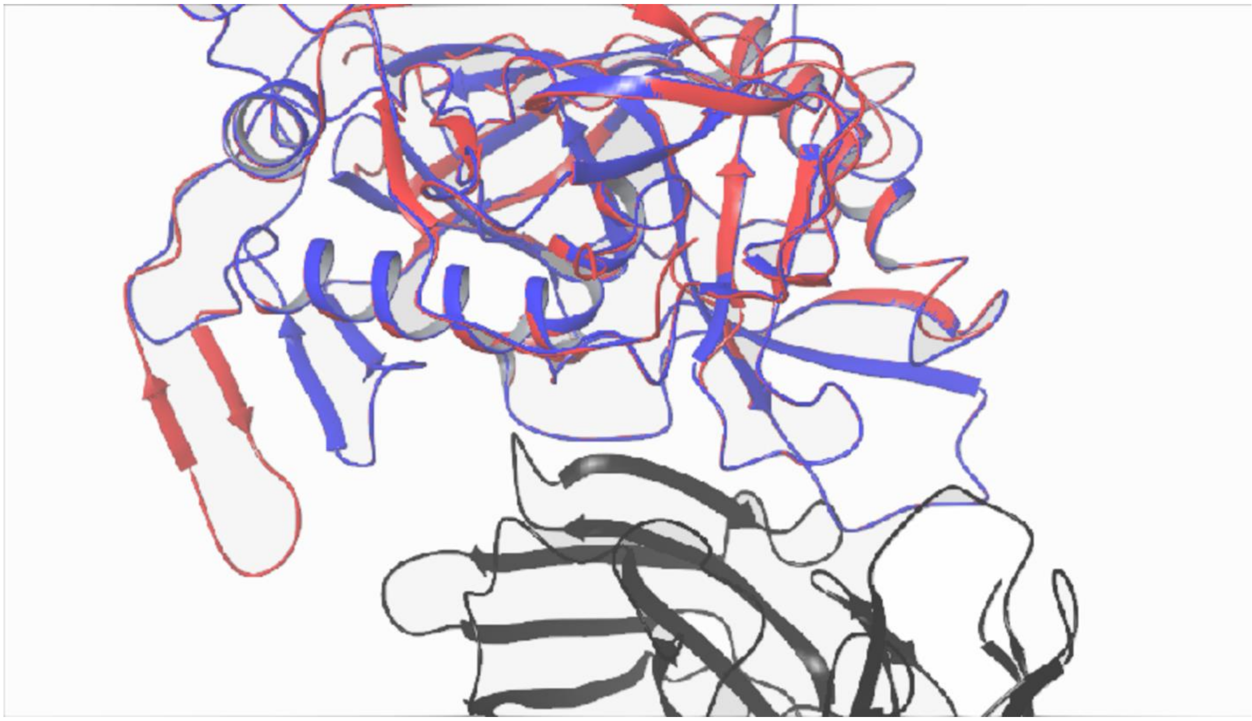

Figure S1: Comparison of crystal structure from PDB ID 3NGB (red) to the RSC3 homology model structure (blue). The antibody is shown in black for reference.

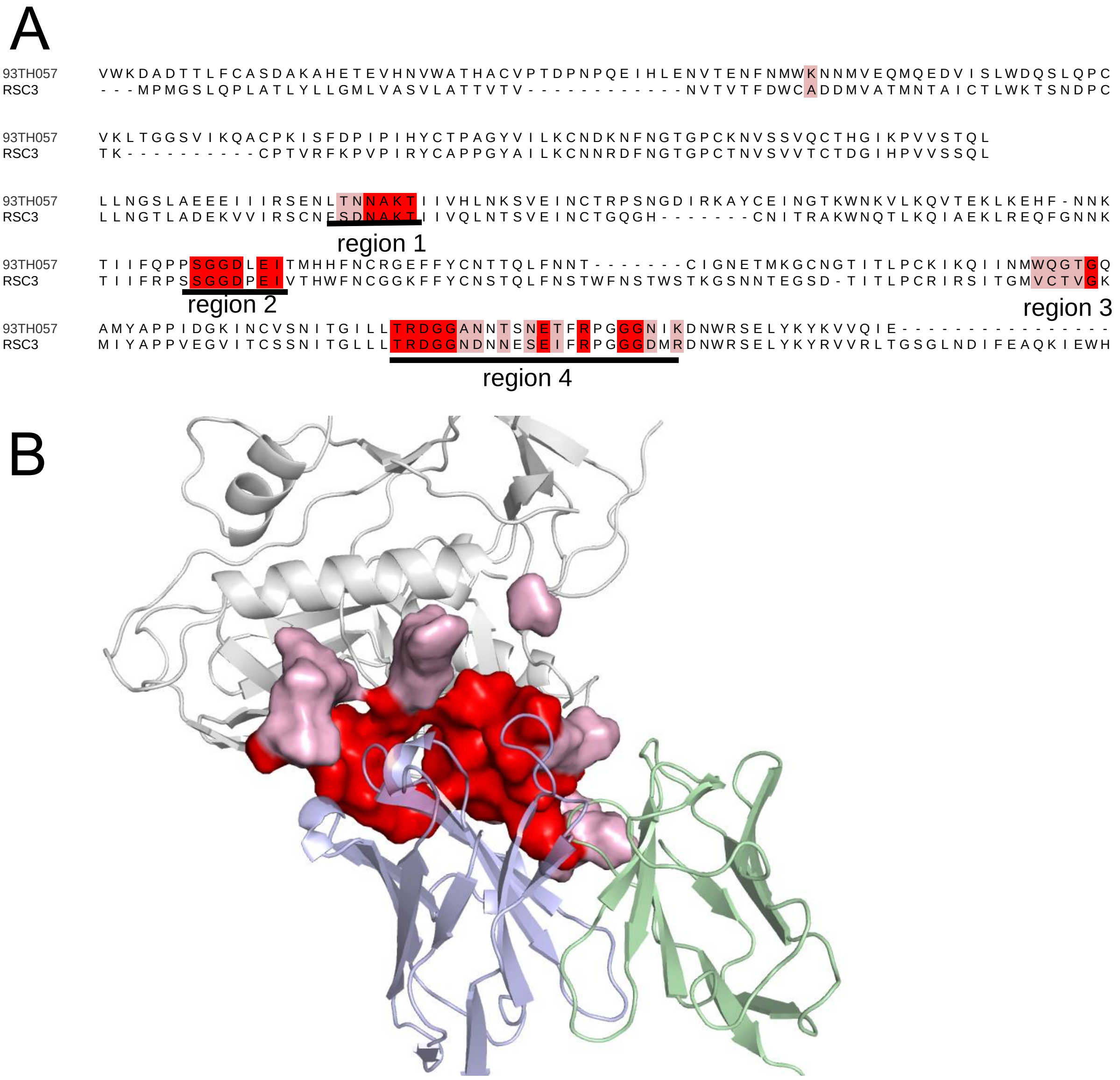

Figure S2: A. Sequence comparison of HIV-1 gp120 from the clade A/E recombinant 93TH057 and resurface stabilized core 3 (RSC3) gp120. 93TH057 as found in the crystallographic structure in complex with VRC01 (pdbID 3NGB) was used as a template for the homology model of RSC3 used in experimental studies of gp120-antibody binding. gp120-Ab interfacial residues that differ between two sequences are highlighted in pink; conserved residues are highlighted in red.

B. Crystal structure of gp120-VRC01 complex. HIV-1 gp120 antibody binding interface is in surface representation with residues colored as in A. gp120 non-intefacial residues are in cartoon representation colored grey. VRC01 antibody is in cartoon representation colored pale blue (heavy chain) and green (light chain).

Homology model stability under molecular dynamics

For each structure a 100ns molecular dynamics (MD) simulation was performed using to assess the stability of the Homology model. The simulations were performed using the same protocol as the fep calculations, with the final fep stage replaced by a MD run of 100ns. For reference, 100ns simulations of the template crystal structure from which the antibody was taken and the RSC3 homology model was built was also run for all three cases. The backbone RMSD from starting structure for the 100ns MD phase is shown in figure S3 for each of the three antibodies for (1) each of the 4 interfacial regions defined in the sequence comparison in figure S2-A (dashed lines), and (2) each of the defined regions on the gp120 plus the whole antibody. The results indicate that the interfacial region remains stable near its initial configuration, and remains stable relative to the antibody.

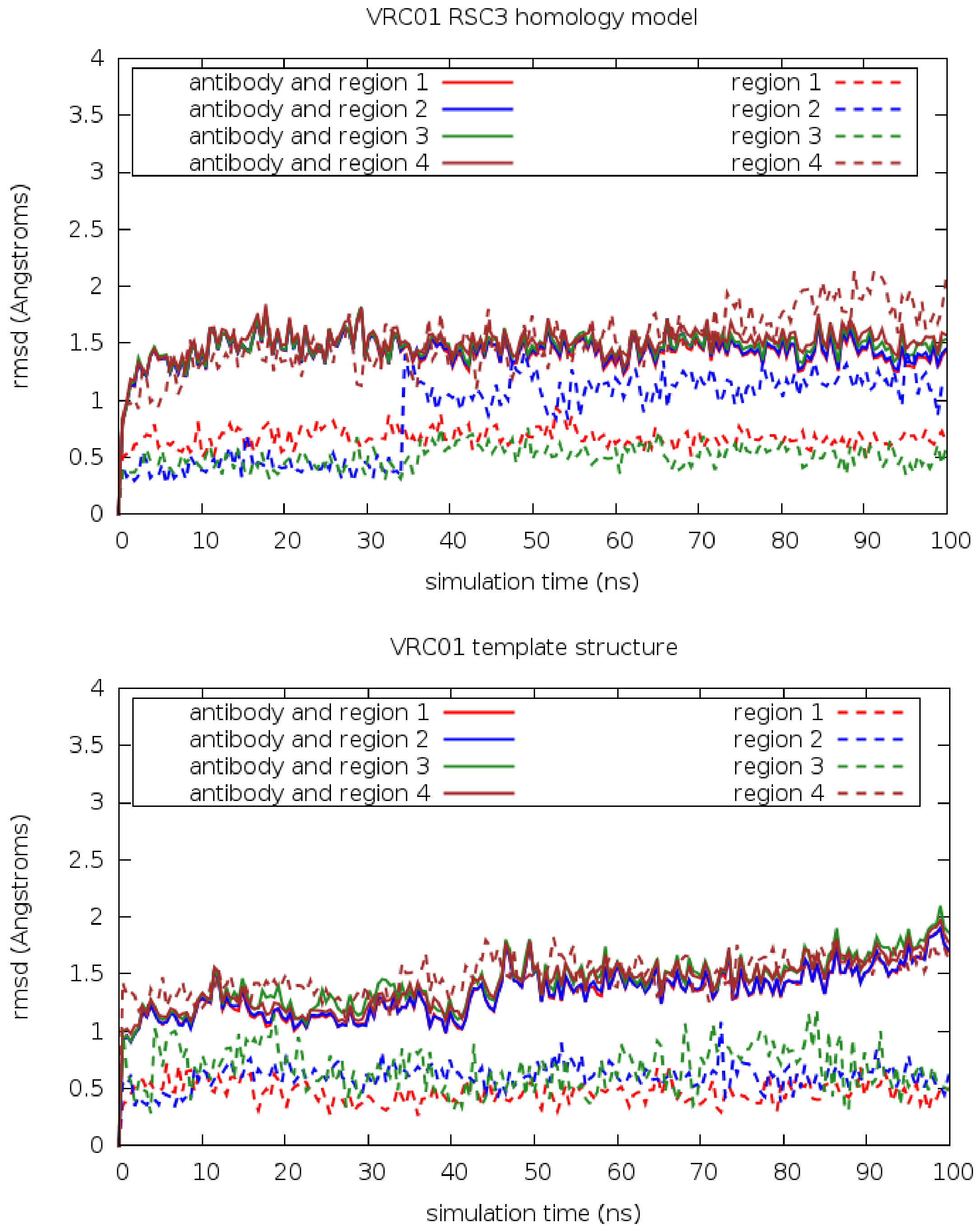
VRC01 RSC3 homology model
antibody and region 1
antibody and region 2
antibody and region 3
antibody and region 4
region 1
region 2
region 3
region 4
rmsd (Angstroms)
simulation time (ns)
VRC01 template structure
antibody and region 1
antibody and region 2
antibody and region 3
antibody and region 4
region 1
region 2
region 3
region 4
rmsd (Angstroms)
simulation time (ns)

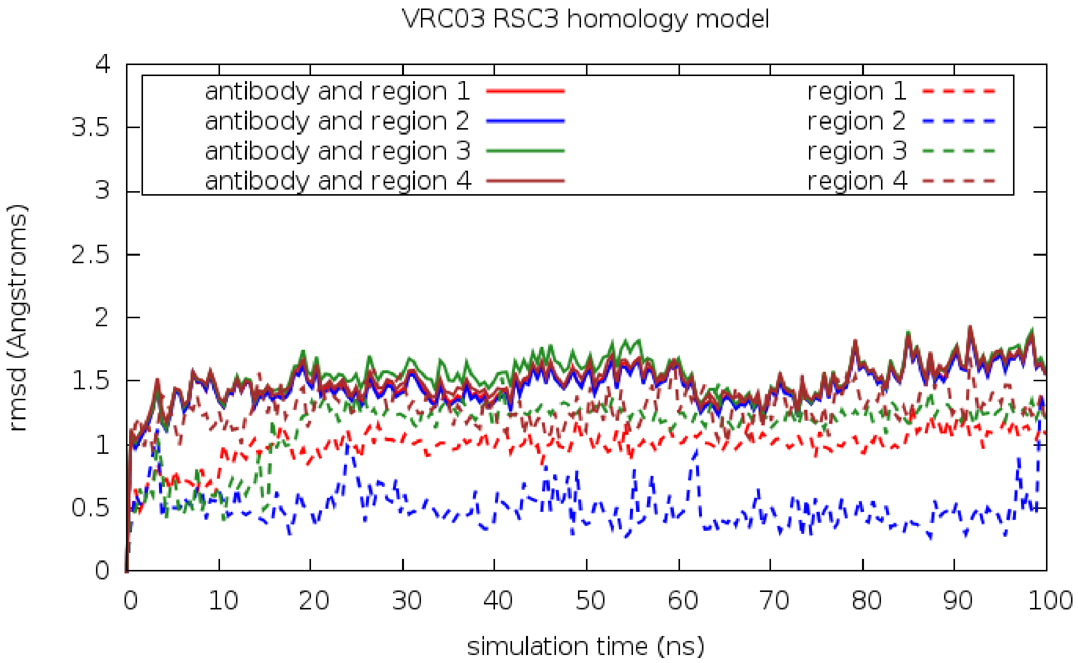

VRC03 RSC3 homology model
antibody and region 1
antibody and region 2
antibody and region 3
antibody and region 4
region 1
region 2
region 3
region 4
rmsd (Angstroms)
simulation time (ns)
0
0.5
1
1.5
2
2.5
3
3.5
4
0
10
20
30
40
50
60
70
80
90
100

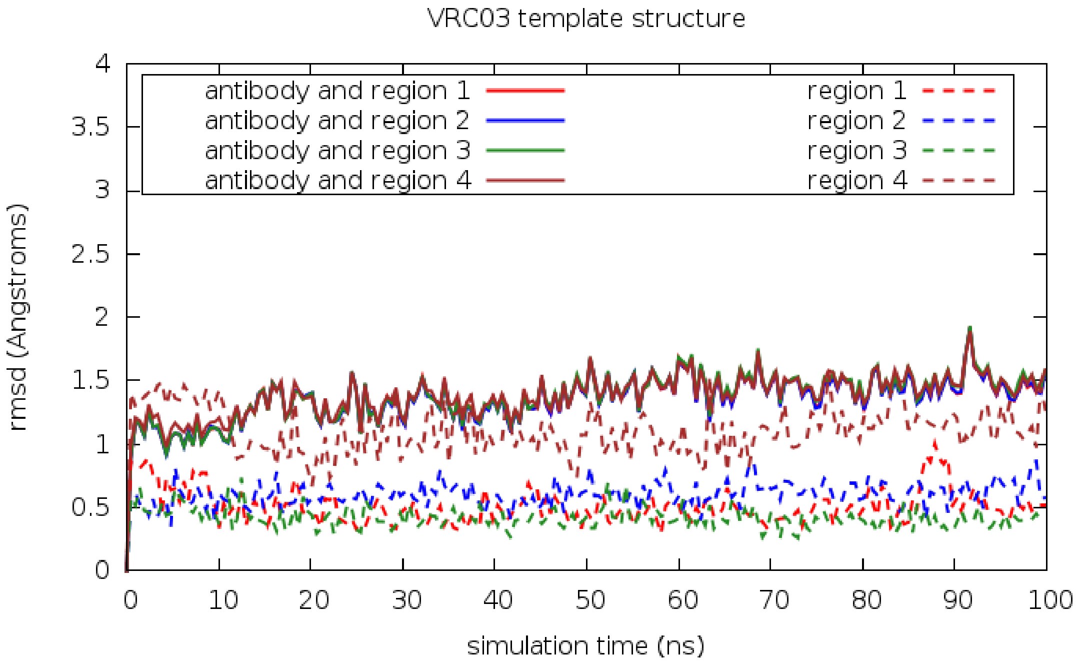

VRC03 template structure
antibody and region 1
antibody and region 2
antibody and region 3
antibody and region 4
region 1
region 2
region 3
region 4
rmsd (Angstroms)
simulation time (ns)
0
0.5
1
1.5
2
2.5
3
3.5
4
0
10
20
30
40
50
60
70
80
90
100

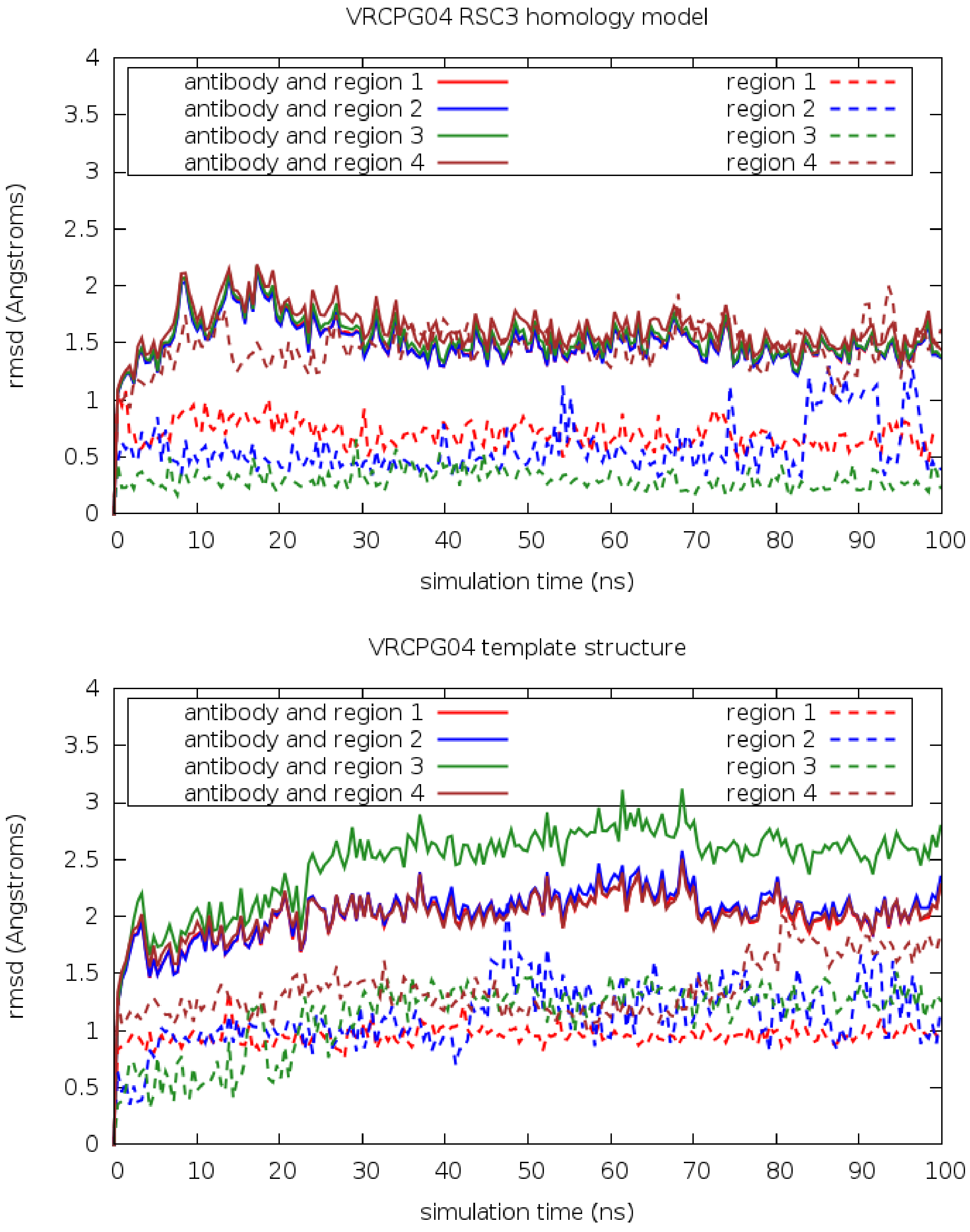

Figure S3: RMSDs of all three bNAb homology models and template crystal structures under MD simulations for the interfacial region of gp120 alone (region 1 –region 4), and for the antibody and the interfacial region (antibody and region 1 – antibody and region 4)

Details of Structure preparation and simulation procedure

The antibody structures were truncated to leave only the domain closest to the antigen. The specific truncation points used are given in table S3. The Antibody structures are prepared by adding hydrogens corresponding to neutral pH and removing glycans and other non-protein constituents The homology model is docked with the antibody crystal structure by aligning with the crystal structure gp120 (the template used to generate it). Several cycles of side chain optimization are performed to re-optimize the structure at the interface.

	heavy chain	light chain
VRC01	V109	V106
VRC03	S112	V106
VRCPG-04	V110	R107

Table S3: Last residue retained from the antibody crystal structure in the truncation scheme used for this study for each bNAb.

All simulations relax the structure with a short sequence of minimization and restrained simulation stages. The FEP/REST phase is performed with 12 windows, with the side chain of the mutating residue included in the hot region. All windows are run for 10ns, unless otherwise noted, using the simulation parameters outlined in the main text in the section on the default protocol. Each 10ns run and all TRP runs were done in duplicate with different initial velocity distributions generated by different random number seeds in the simulation phases.

Effects of REST

To test the effects of the REST scheme, we have performed a significant subset of the cases here without REST. The results presented in table S4 show that the effects of REST is negligible for most cases. There is one case, L34 on the light chain of VRCPG-04, where the use of REST may be reducing the error significantly.

	Experimental ddG Value (kcal/mol)	VRC01 cases (no glyc contacts) seed 0	square error FEP/REST seed 0	VRC01 cases (no glyc contacts) seed 1	square error FEP/REST seed 1	ddG -- no atoms in REST	square error no atoms in REST
W106	4.45	5.25	0.64	3.92	0.28	3.92	0.28
N59	1.76	1.56	0.04	1.18	0.34	0.59	1.37
V58	1.35	0.91	0.19	1.45	0.01	1.08	0.07
W50	1.28	0.82	0.21	-0.15	2.05	0.42	--
N105	1.11	1.55	0.19	0.41	0.49	1.29	0.03
V74	1.08	0.36	0.52	0.55	0.28	0.98	0.01
T33	0.7	0.45	0.06	1.74	1.08	0.82	0.01
Y60	0.54	-0.26	0.64	0.24	0.09	0.92	0.14

M70	0.52	-0.64	1.35	-0.1	0.38	0.19	0.11
Q65	-0.35	0.58	0.86	0.13	0.23	0.63	0.96
Y75	-0.21	0.52	0.53	0.43	0.41	0.51	0.52
I30	-0.01	-0.71	0.49	0.02	0	0.56	0.32
F97	0.85	0.61	0.06	0.87	0	0.46	0.15
Q27	-0.24	-0.03	0.04	0.34	0.34	0.19	0.18
		RMSE:	0.65	RMSE:	0.65	RMSE:	0.57
VRC03							
S58	1.15	0.83	0.1	0.97	0.03	1.39	0.06
Q62	1.11	3.16	4.2	2.49	1.9	3.27	4.65
Y59	0.86	0.34	0.27	0.63	0.05	0.81	0
W54	0.76	2.13	1.88	2.63	3.5	2.58	3.32
Q75	0.09	0.24	0.02	0.25	0.03	0.32	0.05
L53	0.05	0.41	0.13	-0.07	0.01	-0.49	0.29
F97	0.83	0.19	0.41	1.38	0.3	0.47	0.13
		RMSE:	1.00	RMSE:	0.91	RMSE:	1.1
VRCPG-04							
N57	1.53	1.06	0.22	1.02	0.26	2.24	0.51
V52B	-0.82	0.17	0.98	-0.03	0.62	-0.03	0.62
L34	0.57	-0.1	0.45	0.21	0.13	-0.85	2.02
Y98	0.47	0.99	0.27	1.74	1.61	1.25	0.61
G59	0.06	0.61	0.3	0.4	0.12	0.92	0.74
		RMSE:	0.71	RMSE:	0.79	RMSE:	0.95

Table S4: Effects of REST on mutation prediction results is shown. The first two highlighted columns give the default protocol simulation results, and the third the same protocol with no atoms included in the REST hot region. The overall effect of using REST in the cases studied is small. All values are in kcal/mol.

Identification of cases as potential glycan contacts

Since it appears that longer relaxation times may be necessary for glycan contacting cases, in order to assess the ability of the inclusion of the glycan fragment captured in the crystal structure, we limit simulations to cases identified as likely to be affected in the absence of the fragment based upon contacts measured in a molecular dynamics simulation of the glycan-

fragment-containing complex. These simulations used the same set up and equilibration phases as the FEP/REST simulations with the FEP/REST phase replaced by a regular MD production phase.

Inter-atomic contacts were defined by a sigmoidal cutoff function with a cutoff length around 4 Angstroms, $1/\{1+10 \times \exp(d-4)\}$, where d is the inter-atomic distance. This function was used to score the number of interatomic counts between a pair of residues and averaged over frames. The score for each residue was normalized for the number of atoms in the residue. A residue was considered a direct glycan contact if the contact score was at least 3. Potential indirect contacts—residues that might be affected by strong packing with direct contacts—were taken as residues of type TRP, TYR, or PHE with a contact score of at least 15 (two interatomic contacts in the more than half of frames) with directly contacting, or first-layer indirect contacts. Tests on residues of other types meeting the indirect contact criteria, and a few slightly under the threshold were performed and showed no significant effect. For VRC01, no indirect contacts meeting this criteria are found, for VRC03 two indirect contacts are identified, and for VRCPG-04 a relatively large number of potential glycan contacts are identified by this criteria due to contact of the CDR-H3 backbone with the glycan fragment. As can be seen from the overall results, the effect in VRCPG-04 is quite small overall.

FEP calculation data

Individual antibodies

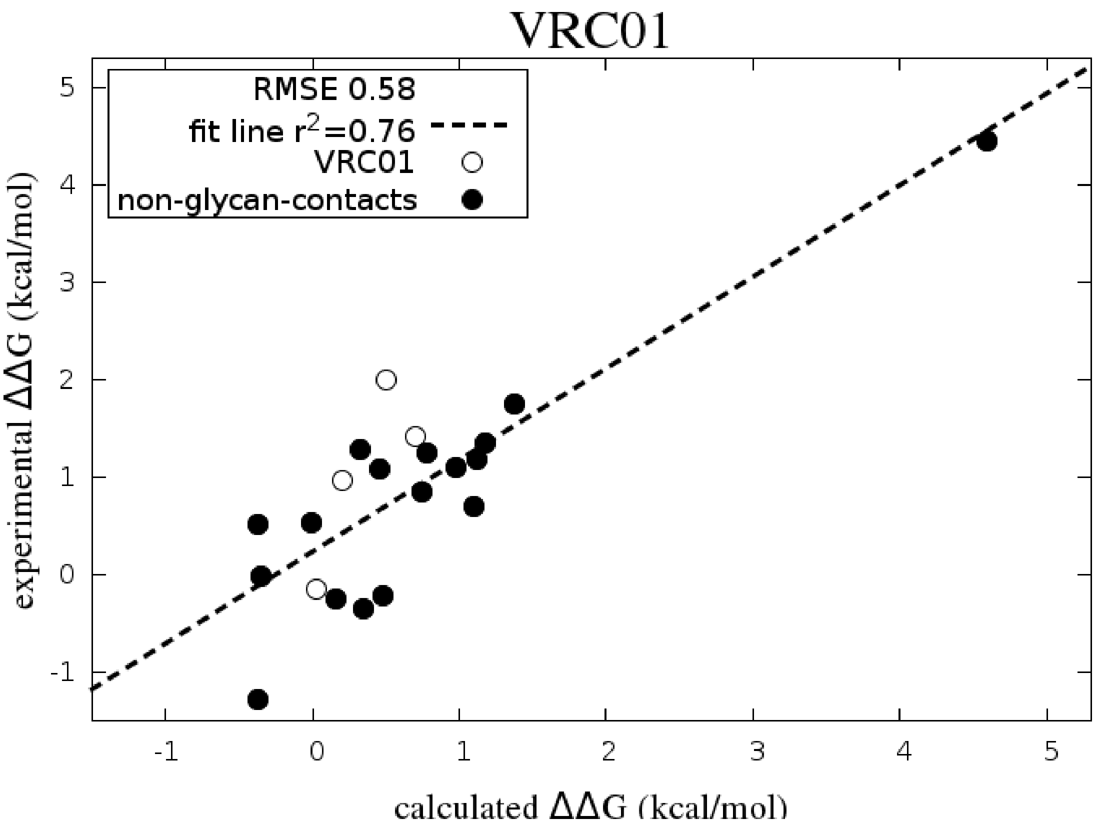

Figure S4: Experimental vs FEP/REST relative binding affinity values for alanine scan cases on VRC01. The correlation coefficient removing the case with the ΔΔG value furthest from the average of the set (noted in parentheses next to the correlation coefficient for the full set) shows that the correlation is not dependent upon the presence of this data point.

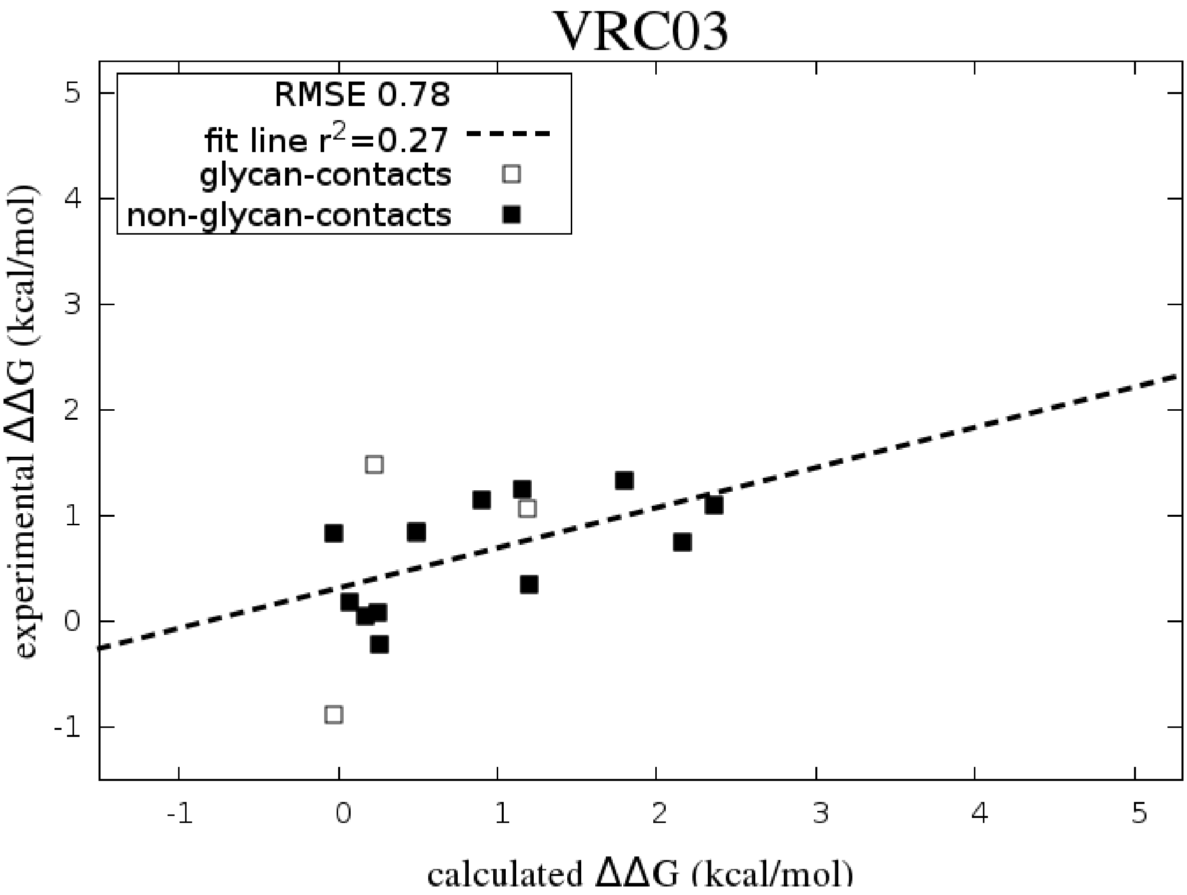

Figure S5: Experimental vs FEP/REST relative binding affinity values for alanine scan cases on VRC03

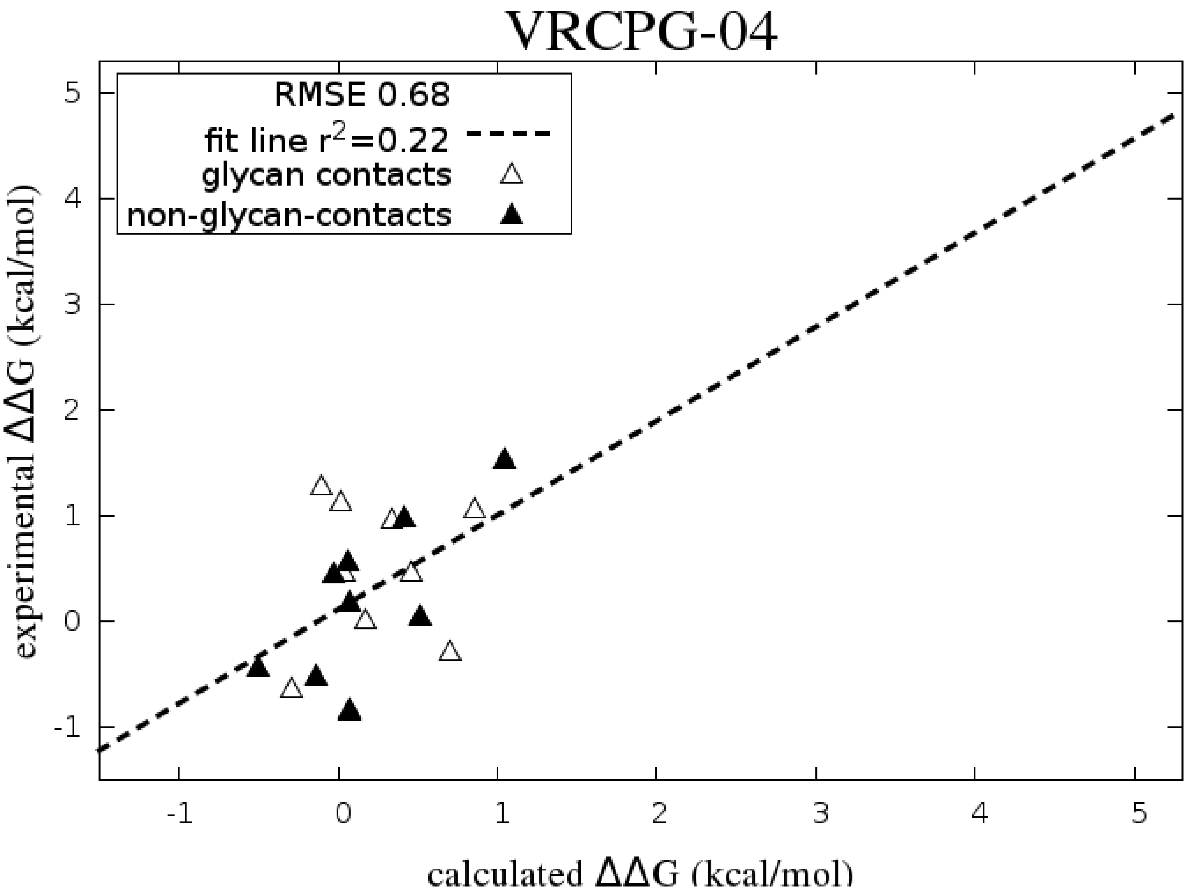

Figure S6: Experimental vs FEP/REST relative binding affinity values for alanine scan cases on VRCPG-04

Summary of data

Table S4 Summarizes all FEP/REST results that were run as part of this study. The result of the default protocol and the result of the revised protocol are presented, and separate 100ns large residue runs, runs seeded with re-predicted loop structures (wild or mutant), and runs performed with glycan fragments present are presented. In cases affected by the suggested protocol modifications, the value included in the final protocol results is highlighted. For cases where multiple trials were performed, the average of these available trials is reported.

bNAb	chain	Mutation	Experiment	default protocol	100ns trial	Using antibody loop prediction	With glycan fragment present	final result in protocol	Change in absolute error from default
VRC01	heavy	W100B	4.45	4.39	**4.59**	--	4.13	4.59	0.07
VRC01	heavy	N58	1.76	1.37	--	--	--	1.37	--
VRC01	heavy	Y100	1.42	0.69	--	--	**0.70**	0.70	-0.01

VRC01	heavy	V57	1.35	1.18	--	--	--	1.18	--
VRC01	heavy	G54	-1.28	-0.59	--	**-0.37**	--	-0.37	0.22
VRC01	heavy	W50	1.28	0.61	**0.33**	--	--	0.33	0.28
VRC01	heavy	G55	1.26	1.99	--	**0.78**	--	0.78	-0.25
VRC01	heavy	W47	1.19	1.71	**1.12**	--	--	1.12	-0.45
VRC01	heavy	N100A	1.11	0.98	--	--	--	0.98	--
VRC01	heavy	V73	1.08	0.46	--	--	--	0.46	--
VRC01	heavy	T33	0.70	1.10	--	--	--	1.10	--
VRC01	heavy	Y59	0.54	-0.01	--	--	--	-0.01	--
VRC01	heavy	M69	0.52	-0.37	--	--	--	-0.37	--
VRC01	heavy	Q64	-0.35	0.35	--	--	--	0.35	--
VRC01	heavy	Y74	-0.21	0.48	--	--	--	0.48	--
VRC01	heavy	I30	-0.01	-0.35	--	--	--	-0.35	--
VRC01	light	Y91	2.01	1.26	--	--	**0.50**	0.50	0.76
VRC01	light	Y28	0.97	-0.63	--	--	**0.20**	0.20	-0.84
VRC01	light	F97	0.85	0.74	--	--	--	0.74	--
VRC01	light	Q27	-0.24	0.16	--	--	--	0.16	--
VRC01	light	S30	-0.15	0.08	--	--	**0.03**	0.03	-0.05
VRC03	heavy	W47	1.48	3.96	-0.05	--	**0.23**	0.23	-1.24
VRC03	heavy	W50	1.33	4.36	**2.68**	--	--	2.68	-1.69
VRC03	heavy	V57	1.25	1.16	--	--	--	1.16	--
VRC03	heavy	S58	1.15	0.90	--	--	--	0.90	--
VRC03	heavy	Q62	1.11	2.36	--	--	--	2.36	--
VRC03	heavy	F100D	1.07	4.48	2.13	--	**1.19**	1.19	-3.30
VRC03	heavy	Y59	0.86	0.49	--	--	--	0.49	--
VRC03	heavy	Y100	0.83	-0.03	--	--	--	-0.03	--
VRC03	heavy	W54	0.76	3.46	**2.16**	--	--	2.16	-1.30
VRC03	heavy	G55	0.35	1.59	--	**1.20**	--	1.20	-0.39
VRC03	heavy	Q75	0.09	0.25	--	--	--	0.25	--
VRC03	heavy	L53	0.05	0.17	--	--	--	0.17	--
VRC03	light	F91	-0.87	1.84	0.68	--	**-0.02**	-0.02	-1.86
VRC03	light	F97	0.83	0.79	--	--	0.49	0.49	0.30
VRC03	light	Q27	-0.22	0.26	--	--	--	0.26	--
VRC03	light	N30	0.20	0.07	--	--	--	0.07	--
VRC-PG04	heavy	N57	1.53	1.04	--	--	--	1.04	--
VRC-PG04	heavy	W50	1.28	2.78	1.57	--	**-0.11**	-0.11	-0.11
VRC-PG04	heavy	W47	1.07	1.18	-1.10	--	**0.86**	0.86	0.09
VRC-PG04	heavy	V56	0.99	0.41	--	--	--	0.41	--

VRC-PG04	heavy	T53	-0.85	0.11	--	--	--		0.07	-0.04
VRC-PG04	heavy	V52B	-0.82	0.07	--	--	--		0.07	--
VRC-PG04	heavy	G100A	-0.63	-0.17	--	--	**-0.30**		-0.30	-0.13
VRC-PG04	heavy	L34	0.57	0.06	--	--	--		0.06	--
VRC-PG04	heavy	Y98	0.47	1.37	--	--	**0.46**		0.46	-0.87
VRC-PG04	heavy	G54	0.46	3.65	--	**-0.03**	--		-0.03	-2.71
VRC-PG04	heavy	T99	-0.43	-0.51	--	--	--		-0.51	--
VRC-PG04	heavy	G100	-0.27	1.12	--	--	**0.70**		0.70	-0.42
VRC-PG04	heavy	G100C	0.19	0.07	--	--	**0.28**		0.28	-0.03
VRC-PG04	heavy	G59	0.06	0.51	--	--	**--**		0.51	--
VRC-PG04	light	Y30	1.13	-0.16	--	--	**0.02**		0.02	-0.18
VRC-PG04	light	L91	0.97	-0.69	--	--	**0.34**		0.34	-1.03
VRC-PG04	light	V3	-0.51	-0.09	--	--	--		-0.14	-0.06
VRC-PG04	light	G31	0.47	-0.14	--	--	**0.04**		0.04	-0.18
VRC-PG04	light	F97	0.02	-0.02	--	--	**0.17**		0.17	0.10

Table S5: Summary of fep cases showing the experimentl value, default protocol value, modified protocol value(s) if any, final value in the revised protocol, and the change in absolute error from the default protocol in cases where the modified protocol applies. All values are in kcal/mol.

Loop prediction control cases

In order to test if loop re-predictions on wild loops could produce a net improvement in results, a subset of cases mostly on the CDR H1 and H2 loops, were chosen for wild type loop re-prediction, and FEP/REST simulation were performed using these starting strucutres.

antibody	Case	ΔΔG experiment	ΔΔG crystal	ΔΔG wild type loop prediction	Change in absolute error	RMSD backbone	RMSD all heavy atoms
VRC03	W55	0.76	2.12	2.16	+0.04	0.09	0.23
VRC03	L54	0.05	0.17	-0.60	0.54	0.09	0.23
VRC-PG04	G57	0.46	3.65	2.96	-0.69	0.23	0.73

VRC-PG04	V52B	-0.82	0.07	0.60	0.53	0.23	0.73
VRC-PG04	T53	-0.85	0.11	1.37	+1.26	0.23	0.73
VRC03	Q63	1.11	2.83	0.03	-0.64	0.16	0.39
VRC01	I30	-0.01	-0.34	0.10	0.02	0.84	1.72
VRC01	T33	0.70	1.10	-0.63	0.93	0.84	1.72

Table S6: The effects on binding affinity prediction of using PLOP to repredict a diverse set of cases in which various wild type residues are mutated to alanine; the RMS error increases slightly, from 1.46 kcal/mole to 1.52 kcal/mol. All values are in kcal/mol.

Error estimation in Calculated Binding affinities:

Average error in the FEP/REST calculations can be estimated from the two independent runs done for each simulation leg. The full width error is estimated from the standard deviation of the four possible DDG values from combining independent trials for each case, and the median full width error is taken as the typical computational error due to noise. The error estimates here include only the cases that used the default protocol value, and the 100ns TRP simulations. Due to computational demands, not all other cases were repeated multiple times.

bNAb	Chain	max, max	min, min	max, min	min, max	error range
W106	heavy	5.25	3.92	5.69	3.48	2.49
N59	heavy	1.18	1.56	1.56	1.18	0.52
V58	heavy	1.38	0.98	1.45	0.91	0.65
W50	heavy	-0.07	0.74	0.82	-0.15	1.22
W47	heavy	1.17	1.08	1.69	0.56	1.09
N105	heavy	0.87	1.09	1.55	0.41	1.12
V74	heavy	0.55	0.36	0.58	0.33	0.30
T33	heavy	1.12	1.07	1.74	0.45	1.24
Y60	heavy	-0.14	0.12	0.24	-0.26	0.54
M70	heavy	-0.27	-0.47	-0.10	-0.64	0.55
Q65	heavy	0.58	0.13	0.68	0.03	0.76
Y75	heavy	0.52	0.43	0.55	0.40	0.17
I30	heavy	-0.40	-0.29	0.02	-0.71	0.71
F97	light	0.84	0.64	0.87	0.61	0.32
Q27	light	-0.03	0.34	0.34	-0.03	0.50
						0.65
VRC03						
W50	heavy	2.98	2.38	2.98	2.29	0.88
V57	heavy	1.19	1.13	1.23	1.09	0.15
S58	heavy	0.97	0.83	0.97	0.77	0.24

Q62	heavy	2.97	2.68	2.97	2.49	0.56
Y59	heavy	0.63	0.34	0.63	0.10	0.60
Y100	heavy	-0.08	0.02	0.42	-0.48	0.88
W54	heavy	2.63	2.13	2.63	2.09	0.71
Q75	heavy	0.25	0.24	0.25	0.24	0.01
L53	heavy	0.20	0.14	0.27	-0.07	0.35
F97	light	0.87	0.70	1.38	0.19	1.16
Q27	light	0.28	0.23	0.41	0.10	0.30
N30	light	0.07	0.06	0.07	-0.04	0.13
						0.45
VRCPG-04						
N57	heavy	1.02	1.06	1.02	0.95	0.11
V56	heavy	0.50	0.32	0.50	0.25	0.29
T53	heavy	0.82	-0.79	0.82	-1.08	2.41
V52B	heavy	0.17	-0.03	0.31	-0.14	0.47
G100A	heavy	-0.10	-0.23	0.00	-0.39	0.40
L34	heavy	0.00	0.11	0.21	-0.10	0.32
G59	heavy	0.61	0.40	0.66	0.35	0.36
Y30	light	-0.12	-0.20	0.00	-0.20	0.22
V3	light	-0.14	-0.03	0.06	-0.20	0.27

Table S7: As an estimate on the average error in the FEP/REST calculations, the four possible DDG values that could be obtained from the two independent runs for each of the bound complex and unbound antibody legs. Additional trials for legs whose initial 10ns trials differed by more than 0.5 kcal are not included in this analysis. All values are in kcal/mol.
